# Simultaneous fluorescence imaging of hydrogen peroxide in mitochondria and endoplasmic reticulum during apoptosis†
†Electronic supplementary information (ESI) available: Detailed experimental procedures, characterization of compounds, some fluorescence images. See DOI: 10.1039/c6sc01793b


**DOI:** 10.1039/c6sc01793b

**Published:** 2016-06-01

**Authors:** Haibin Xiao, Ping Li, Xiufen Hu, Xiaohui Shi, Wen Zhang, Bo Tang

**Affiliations:** a College of Chemistry, Chemical Engineering and Materials Science , Collaborative Innovation Center of Functionalized Probes for Chemical Imaging in Universities of Shandong , Key Laboratory of Molecular and Nano Probes , Ministry of Education , Shandong Provincial Key Laboratory of Clean Production of Fine Chemicals , Shandong Normal University , Jinan 250014 , P. R. China . Email: tangb@sdnu.edu.cn

## Abstract

Cell apoptosis is a biochemical and molecular pathway essential for maintaining cellular homeostasis. It is an integrated process involving in a series of signal transduction cascades. Moreover, the apoptotic pathways may be initiated inside various subcellular organelles. Increasing evidence indicates that hydrogen peroxide (H_2_O_2_) is closely related to cell apoptosis, particularly in the mitochondria. However, during the apoptotic process, the synergetic variation of H_2_O_2_ levels in different compartments is seldom explored, particularly in two important organelles: mitochondria and endoplasmic reticulum (ER). To solve this problem, we developed two new organelle-specific fluorescent probes termed **MI-H_2_O_2_** and **ER-H_2_O_2_** that can detect H_2_O_2_ in mitochondria and ER, respectively or simultaneously. Experimental results demonstrated that **MI-H_2_O_2_** and **ER-H_2_O_2_** display distinguishable excitation and emission spectra, as well as excellent organelle targeting capabilities. Therefore, we used **MI-H_2_O_2_** and **ER-H_2_O_2_** to successfully image exogenous or endogenous hydrogen peroxide in the mitochondria and ER. Interestingly, during diverse apoptotic stimuli, dual-color fluorescence imaging results revealed that the changes of H_2_O_2_ levels in mitochondria and ER are different. The H_2_O_2_ levels are enhanced in both the mitochondria and ER during the l-buthionine sulfoximine (BSO)-treated cell apoptosis process. During mitochondria-oriented apoptosis induced by carbonyl cyanide *m*-chlorophenylhydrazone (CCCP) or rotenone, H_2_O_2_ levels prominently and continuously increase in the mitochondria, whereas the ER H_2_O_2_ levels were found to rise subsequently after a delay. Moreover, during ER-oriented apoptosis induced by tunicamycin, ER is the major site for overproduction of H_2_O_2_, and delayed elevation of the H_2_O_2_ levels was found in the mitochondria. Altogether, this dual-probe and multicolor imaging approach may offer a proven methodology for studying molecular communication events on H_2_O_2_-related apoptosis and also other physiological and pathological processes within different subcellular organelles.

## Abstract 2

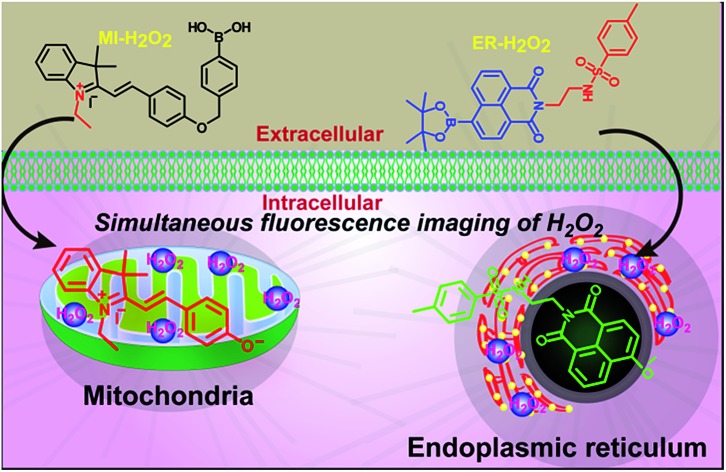
We have developed two new organelle-specific fluorescent probes for the simultaneous imaging of hydrogen peroxide in the mitochondria and the endoplasmic reticulum during apoptosis.

## Introduction

Apoptosis, a mode of programmed cell death, is critical for maintaining cellular homeostasis. Notably, cell apoptosis is an integrated mechanism involving in a series of signal transduction cascades and the synergistic effects of subcellular organelles.^1^,^2^ There are many different pathways related to apoptosis including ligation of plasma membrane death receptors (the ‘extrinsic’ pathway) and perturbation of intracellular homeostasis (the ‘intrinsic’ pathway). Many organelles are major sites of integration of pro-apoptotic signaling, or damage sensing, and can sense stressful and pathogenic alterations to initiate cell apoptosis.^3^,^4^ In particular, mitochondria-elicited and endoplasmic reticulum (ER) stress-regulated apoptosis are of wide concern. However, the interaction and interplay, as well as synergistic variations of the corresponding signal molecules, during mitochondria-triggered and ER-triggered apoptosis have not been elucidated. H_2_O_2_, as one type of reactive oxygen species (ROS) is an inevitable byproduct of cell metabolism and a common marker and signal molecule for oxidative stress that is associated with apoptosis.^5^–^8^ Therefore, research on H_2_O_2_ biology will play a vital role in revealing the relationship between cell apoptosis and H_2_O_2_ as well as molecular communication within different organelle-derived apoptosis processes (signal pathways). For instance, H_2_O_2_ is generated in mitochondria as an early major mediator in rotenone-induced or ceramide-induced apoptosis.^9^,^10^ And *vice versa*, H_2_O_2_ can induce apoptosis *via* a mitochondria-related apoptotic pathway.^3^,^11^,^12^ Excessive, or inaccurate, protein folding in the ER may lead to ER stress, and chronic or unresolved ER stress can trigger cell apoptosis, which is responsible for a significant proportion of H_2_O_2_ elevation.^13^–^17^ However, due to lack of ideal tools, the cooperation of mitochondria and ER in H_2_O_2_ biology during apoptosis has not been interpreted. In particular, when cells initiate the apoptotic cascades through one subcellular organelle under certain stimulation, the changes of H_2_O_2_ levels in the other involved organelles have not been studied up to now, which impedes the better understanding of H_2_O_2_-related physiology and pathology in apoptosis within different subcellular organelles. As a consequence, there is an urgent need to exploit new approaches for simultaneously visualizing H_2_O_2_ levels in different organelles sensitively and selectively.

Fluorescent probes are well-suited tools to map the spatial and temporal distribution of interested species within living cells.^18^–^21^ In particular, simultaneous fluorescence imaging of an active molecule in different subcellular organelles will facilitate understanding of complicated chemical and biological processes because this approach, with real time operation, can offer reliable data. Motivated to meet this need, specific conditions must be considered: (1) the probes must possess outstanding organelle-targeting abilities; (2) the probes must display distinguishable excitation or emission spectra that can be monitored simultaneously with multicolor confocal fluorescence imaging. In recent years, mounting synthetic fluorescent H_2_O_2_ indicators has been developed, which greatly contributes to the understanding of H_2_O_2_ biological chemistry. Among them, a series of organelle-targeting fluorescent probes have been exploited, especially for the mitochondria,^22^–^29^ the lysosome,^30^–^33^ and the nucleus.^34^,^35^ However, no report in which simultaneous fluorescence imaging of hydrogen peroxide in different organelles, such as the mitochondria and ER, was presented and utilized to study the H_2_O_2_-related chemical events up to now.

**Scheme 1:**
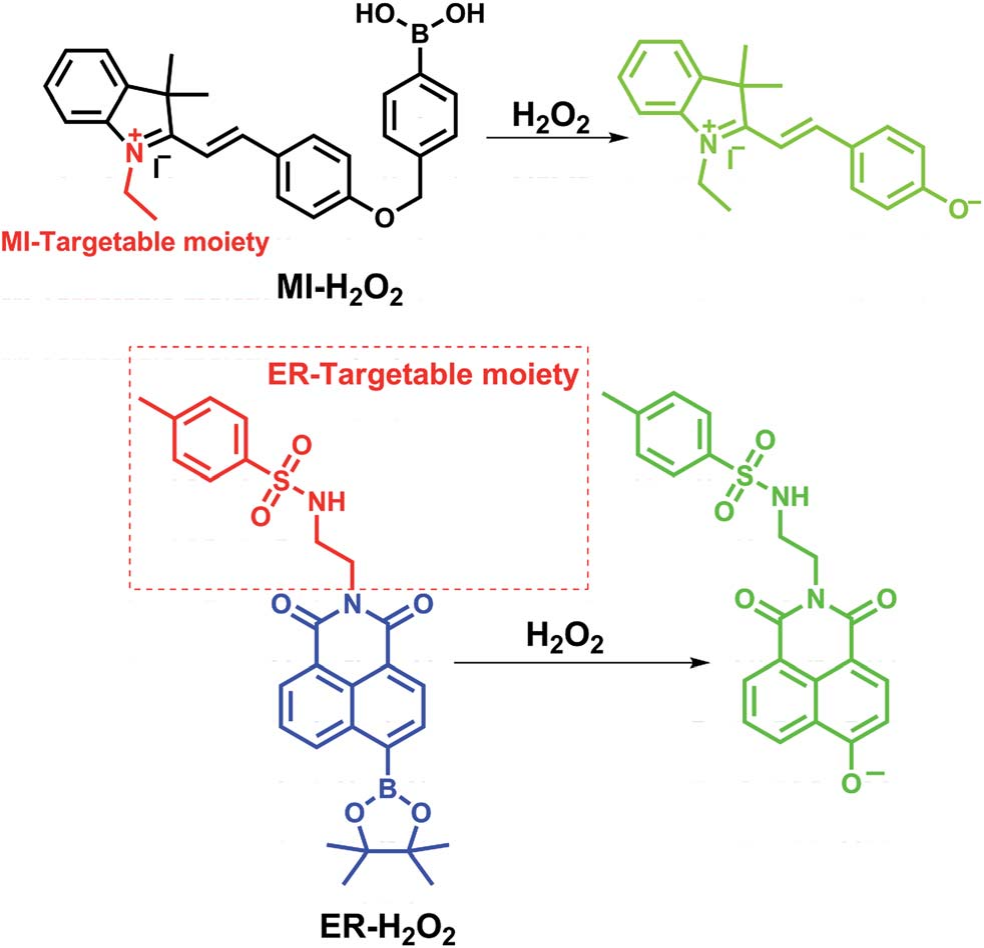
The chemical structures of **MI-H_2_O_2_** and **ER-H_2_O_2_** and corresponding response mechanism.

In this study, we reported the synthesis and application of two H_2_O_2_-selective fluorescent probes, termed **MI-H_2_O_2_** and **ER-H_2_O_2_**, based on a boronic acid/ester deprotection^36^–^40^ mechanism (Scheme 1). **MI-H_2_O_2_** and **ER-H_2_O_2_** can preferentially accumulate in the mitochondria and ER, respectively, because of the lipophilic cationic and methyl sulphonamide moieties, respectively.^41^,^42^ Additionally, they display obviously discriminable spectra that benefits live cell multicolor imaging. **MI-H_2_O_2_** is comprised of merocyanine as a fluorescence reporter and boronic acid as a specific masking group. The boronic acid group can be removed upon reaction with hydrogen peroxide and converted to an oxygen anion, forming an intense push–pull conjugated system that fluoresces strongly (Scheme 1). Meanwhile, **ER-H_2_O_2_** consists of a 1,8-naphthalimide fluorophore and a boronic ester recognition group. After reaction of H_2_O_2_, an electron donor and an acceptor conformation was engendered, and that has been continually used in ratiometric fluorescent probes owing to its outstanding internal charge transfer (ICT) structure. As expected, our results indicated that **MI-H_2_O_2_** and **ER-H_2_O_2_** can visualize exogenous or endogenous H_2_O_2_ in mitochondria and ER, respectively. Also, simultaneous multicolor fluorescence imaging utilizing these two probes revealed that H_2_O_2_ levels changed differentially in the mitochondria and the ER during different apoptotic stimuli.

## Results and discussion

### Chemical synthesis

The target probes **MI-H_2_O_2_** and **ER-H_2_O_2_** were composed of three parts, *i.e.*, fluorophores, recognition units, and organelle-targeting moieties (Scheme 1). The fluorophores were constructed based on the merocyanine and 1,8-naphthalimide scaffolds due to their outstanding photophysical characteristics and easy preparation. The recognition units were universal boronic acid/ester and the lipophilic cationic and methyl sulphonamide served as the mitochondria and ER targeting moieties, respectively. The detailed synthetic routes of **MI-H_2_O_2_** and **ER-H_2_O_2_** are provided in the ESI.† All the compounds and probes were characterized by ^1^H NMR, ^13^C NMR and HR-MS.

### *In vitro* optical properties of **MI-H_2_O_2_** and **ER-H_2_O_2_** to H_2_O_2_

**Fig. 1:**
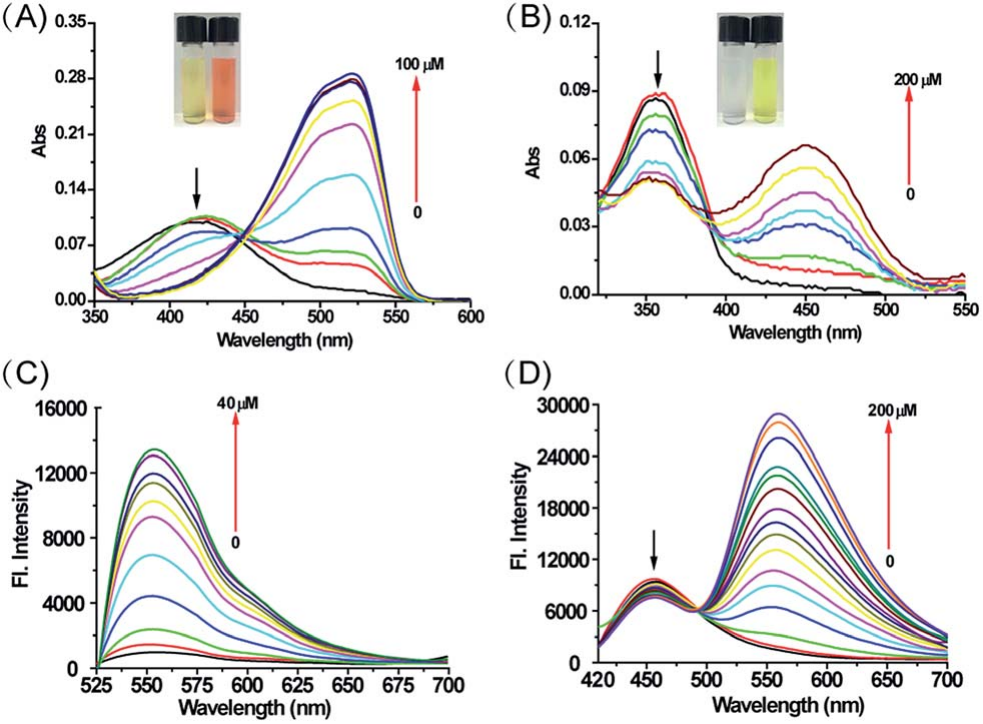
The absorption and fluorescence spectra changes of **MI-H_2_O_2_** (A and C) and **ER-H_2_O_2_** (B and D) to H_2_O_2_. The excitation wavelength was 525 nm or 400 nm for **MI-H_2_O_2_** and **ER-H_2_O_2_**, respectively. Inset: color changes of **MI-H_2_O_2_** and **ER-H_2_O_2_** solution (50 μM) in the absence or presence of H_2_O_2_ (500 μM).

To understand the effect of H_2_O_2_ on the photophysical properties of **MI-H_2_O_2_** and **ER-H_2_O_2_**, we examined the absorption and fluorescence emission spectra of **MI-H_2_O_2_** and **ER-H_2_O_2_** in the absence and presence of H_2_O_2_. As shown in Fig. 1A, **MI-H_2_O_2_** was almost colorless with an absorption maximum at 425 nm. Upon addition of H_2_O_2_, the solution turned to light pink with increasing absorption bands at 525 nm. For **ER-H_2_O_2_**, its solution changed from colorless to yellow, and the absorption red-shifted from 360 nm to 460 nm (Fig. 1B). This demonstrated these two probes can serve as “naked-eye” sensors for colorimetric detection of H_2_O_2_. We subsequently investigated the fluorescence responses of **MI-H_2_O_2_** and **ER-H_2_O_2_** to H_2_O_2_ under physiological conditions (10 mM PBS, pH 8.0 for **MI-H_2_O_2_** and 7.4 for **ER-H_2_O_2_**). For **MI-H_2_O_2_**, in the presence of H_2_O_2_, the fluorescence emission peak at 555 nm increased dramatically when excited at 525 nm (Fig. 1C). It showed an about 13-fold fluorescence enhancement upon addition of 40 μM H_2_O_2_, and the fluorescence quantum yield increased from 0.0087 to 0.11. Moreover, two linear regressions were obtained between the fluorescence intensity at 555 nm and the H_2_O_2_ concentration in the range of 15–40 μM and 0.5–15 μM with a detection limit of 80 nM (Fig. S1†). For **ER-H_2_O_2_**, upon addition of H_2_O_2_, the maximum emission peak exhibited a 100 nm red shift from about 458 nm to 558 nm when excited at 400 nm, which makes **ER-H_2_O_2_** suitable for ratiometric detection due to the introduction of an electron-donating oxygen anion (Fig. 1D). It displayed an about 19-fold fluorescence enhancement at 558 nm upon addition of 200 μM H_2_O_2_, and the fluorescence quantum yield increased from 0.007 to 0.24. The ratio *F*_558_/*F*_458_ increased from 0.161 to 3.639 in the absence and presence of H_2_O_2_ (200 μM). There was good linearity between the fluorescence intensity ratio, *F*_558_/*F*_458_, and the concentrations of H_2_O_2_ in the range of 0 to 40 μM with a detection limit of 120 nM (Fig. S2†). The detection limit of **MI-H_2_O_2_** and **ER-H_2_O_2_** was favorable and comparable to many previously reported H_2_O_2_ sensors.^43^ All these results indicated that **MI-H_2_O_2_** and **ER-H_2_O_2_** could detect H_2_O_2_ with excellent sensitivity.

### The selectivity and reaction kinetics

Afterwards we evaluated the selectivity of these two probes towards other ROS and metal ions. As illustrated in Fig. S3 and S4,† almost no fluorescence intensity and ratio changes were observed in the presence of NaClO, OH˙, O_2_˙^–^, TBHP, ONOO^–^, and other common metal ions for **MI-H_2_O_2_** and **ER-H_2_O_2_**. In addition, the response kinetic studies for the reactions and photostable experiments were examined in Fig. S5 and S6.† The pseudo-first-order rate constants were determined as *k*′ = 4.35 × 10^–3^ S^–1^ and 2.35 × 10^–3^ S^–1^ for the reaction of **MI-H_2_O_2_** and **ER-H_2_O_2_** with 100 equiv. of H_2_O_2_ respectively. All of the above experiments imply that both **MI-H_2_O_2_** and **ER-H_2_O_2_** possess high selectivity and reaction speed to H_2_O_2_ as well as excellent photostability before and after reaction with H_2_O_2_.

### Organelle-targeting ability of **MI-H_2_O_2_** and **ER-H_2_O_2_**

**Fig. 2:**
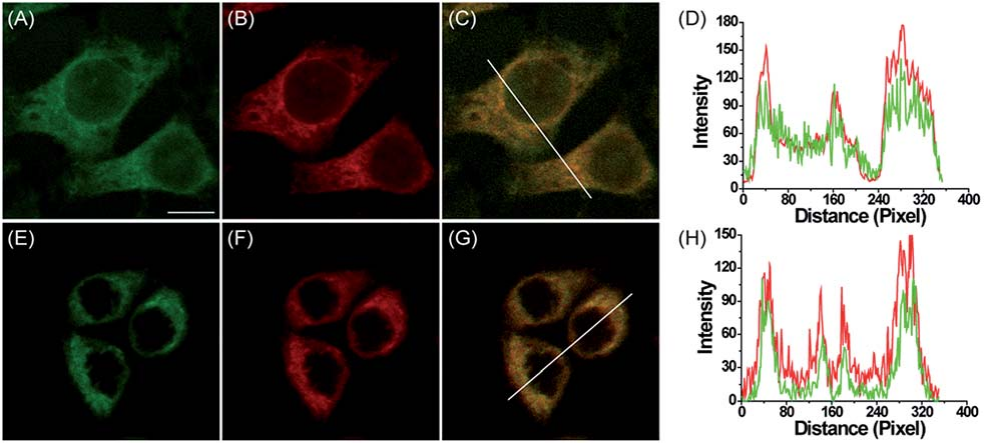
Confocal fluorescence images of HepG2 cells stained with **MI-H_2_O_2_** or **ER-H_2_O_2_** with corresponding commercial organelle-specific dyes. (A) Fluorescence image of **MI-H_2_O_2_** (10 μM, green channel, Ex = 514 nm, collected 520–580 nm) in cells pretreated with 200 μM H_2_O_2_ for 60 min. (B) Fluorescence image of Mito-Tracker Deep Red (0.5 μM, red channel, Ex = 633 nm, collected 640–700 nm). (C) Overlay of (A) and (B). (D) Intensity profile of the white line in image C. (E) Fluorescence image of **ER-H_2_O_2_** (10 μM, green channel, Ex = 405 nm, collected 500–620 nm) in cells pretreated with 200 μM H_2_O_2_ for 60 min. (F) Fluorescence image of ER-Tracker Red (0.5 μM, red channel, Ex = 543 nm, collected 580–630 nm). (G) Overlay of (E) and (F). (H) Intensity profile of the white line in image G. Scale bar: 10 μm.

Subsequently, the subcellular distribution of **MI-H_2_O_2_** and **ER-H_2_O_2_** in HepG2 cells were studied. As shown in Fig. 2A–D, fluorescence of **MI-H_2_O_2_** was well co-localized with that of Mito-Tracker Deep Red (overlap coefficient 0.90), the commercial mitochondria-specific dye, which demonstrated that **MI-H_2_O_2_** exhibited excellent mitochondria-targeting abilities that are attributable to the lipophilic cationic merocyanine moiety. At the same time, the commercial specific staining probe ER-Tracker Red was used to co-stain live cells with **ER-H_2_O_2_**. As displayed in Fig. 2E–H, the fluorescence of **ER-H_2_O_2_** was well co-localized with that of ER-Tracker Red with an overlap coefficient of 0.91. The results verified our design and implied a preferential distribution of **MI-H_2_O_2_** and **ER-H_2_O_2_** into the mitochondria and ER, respectively. Furthermore, the intrinsic organelle-targeting ability of **MI-H_2_O_2_** and **ER-H_2_O_2_** was investigated in 4T1 cells. A consistent and perfect targeting capability was found (Fig. S7†). In addition, the MTT assay indicated **MI-H_2_O_2_** and **ER-H_2_O_2_** had low cytotoxicity (Fig. S8†).

**Fig. 3:**
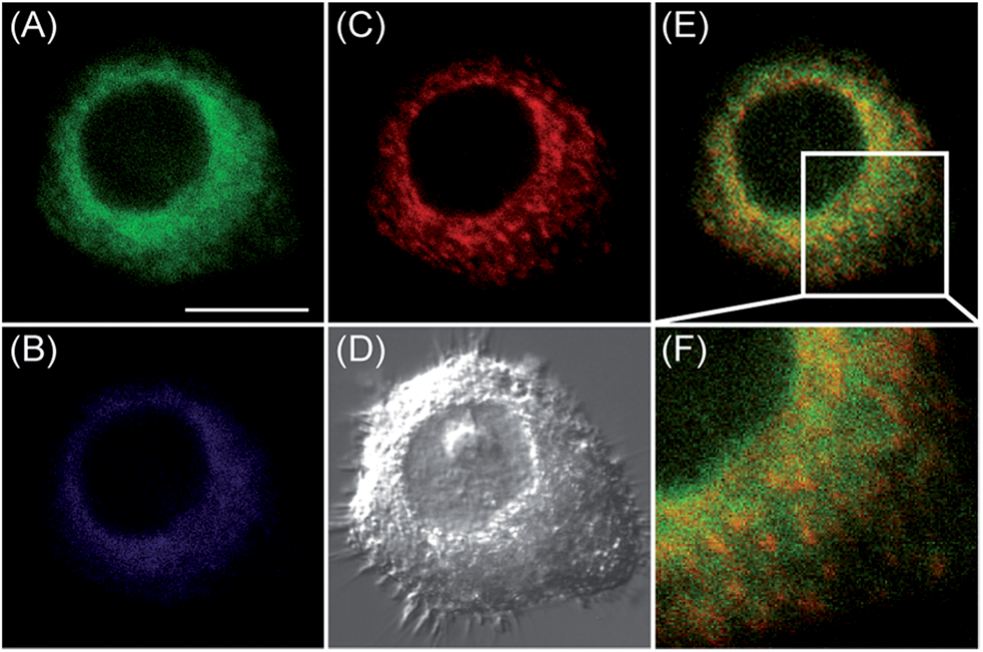
Confocal fluorescence images of live HepG2 cells stained simultaneously with **ER-H_2_O_2_** and **MI-H_2_O_2_** (10 μM). **ER-H_2_O_2_** was excited by 405 nm, and collected at 500–620 nm for the green channel (image A) and at 430–470 nm for the blue channel (image B). **MI-H_2_O_2_** was excited by 543 nm, and collected at 550–600 nm for the red channel (image C). (D) was the bright-field image. (E) was overlay of (A) and (C). (F) was the enlarged image from the square marked in image (E). Scare bar: 20 μm.

With these two excellent organelle-targeting probes in hand, we next intended to explore their optical performance in live cells. To completely avoid the spectral overlap for confocal fluorescence imaging, we chose 543 nm as excitation wavelength for **MI-H_2_O_2_**. It is because **ER-H_2_O_2_** cannot be excited at 543 nm, but can be easily excited by 405 nm. In stark contrast, fluorescence enhancement of **MI-H_2_O_2_** by H_2_O_2_ can be readily excited by 543 nm but not 405 nm (Fig. S9†). This enabled multicolor fluorescence imaging simultaneously utilizing **ER-H_2_O_2_** and **MI-H_2_O_2_** by excitation wavelengths of 405 and 543 nm, respectively. Therefore, we performed the confocal fluorescence imaging of H_2_O_2_ in live cells with both **ER-H_2_O_2_** and **MI-H_2_O_2_**. After treating with 200 μM H_2_O_2_ for 1 h, HepG2 cells were incubated with **ER-H_2_O_2_** and **MI-H_2_O_2_** for 1 h, and the probes can fluoresce well under excitation of 405 and 543 nm. As shown in Fig. 3E, there is basically no overlap fluorescence between the green channel of **ER-H_2_O_2_** (image A, excited by 405 nm and collected at 500–620 nm) and the red channel of **MI-H_2_O_2_** (image C, excited by 543 nm and collected at 550–600 nm). An enlarged image from Fig. 3E indicated it indeed exhibited remarkable intensity difference in these two images (Fig. 3F). All the date established **ER-H_2_O_2_** and **MI-H_2_O_2_** have differentiable excitation and emission spectra and enable multicolor fluorescence imaging of exogenous H_2_O_2_ in the mitochondria and the ER simultaneously.

### Endogenous H_2_O_2_ imaging in live cells with **MI-H_2_O_2_** and **ER-H_2_O_2_**

We next visualized the endogenous H_2_O_2_ in live cells induced by different stimulus in the mitochondria and the ER respectively. Phorbol 12-myristate 13-acetate (PMA) may trigger production of ROS in mitochondria via activating of PKC (protein kinase C).^44^*via* The fluorescence of **MI-H_2_O_2_** increased obviously upon addition of PMA for 60 min (Fig. S10A†). Tunicamycin (Tm) produced by several bacteria, is known to inhibit glycosylation during protein or glycolipid synthesis, which can cause the accumulation of proteins or lipids in the ER, leading to acute ER stress.^45^ To image the endogenous production of H_2_O_2_ in live cells by utilizing **ER-H_2_O_2_** in this condition, we performed the ratiometric fluorescence imaging of Tm-treated HepG2 cells. As illustrated in Fig. S10B,† Tm-treated (10 μg mL^–1^, 8 h) cells showed obviously bright fluorescence in the green channel compared to that of DMSO-treated cells (Fig. S10B6 and B2†). The ratiometric image between the two channels (green to blue) further validated that H_2_O_2_ was produced in this Tm-induced ER stress model (Fig. S10B7 and B3†). The similar results were observed in the mouse 4T1 cells (Fig. S11†). In addition, **ER-H_2_O_2_** was utilized to detect the production of H_2_O_2_ in live cells treated with dithiothreitol (DTT), a reducing agent that can disturb the formation of protein disulfide bonds and induce ER stress.^46^,^47^ The results presented in Fig. S12† displayed that there was an obvious fluorescence increase in the green channel, indicating a rise in the H_2_O_2_ concentration. Furthermore, to explore whether there is a burst of H_2_O_2_ concentration in live cells treated with anticancer drugs targeting the ER, **ER-H_2_O_2_** was applied for fluorescence imaging in live HepG2 cells treated with nelfinavir, a lead HIV protease inhibitor and broad-spectrum anticancer drug that can lead to ER stress.^48^–^50^ As shown in Fig. S13,† upon treated with 200 μg mL^–1^ nelfinavir for 160 min, the fluorescence intensity of the green channel increased (Fig. S13B and F†) and the ratio between the green channel and the blue channel was higher (Fig. S13C and G†). Additionally, the cells showed shrinking morphological changes (Fig. S13D and H†), indicating the apoptosis of cells involved in the ER-stress pathway. At the same time, a similar experimental result was observed in the nelfinavir-induced 4T1 cells (Fig. S14†). Collectively, all this data demonstrated that **MI-H_2_O_2_** and **ER-H_2_O_2_** can image endogenous H_2_O_2_ well in the corresponding subcellular organelles under different stimuli.

### Simultaneous fluorescence imaging of H_2_O_2_ with **MI-H_2_O_2_** and **ER-H_2_O_2_** during apoptosis with different stimuli

**Fig. 4:**
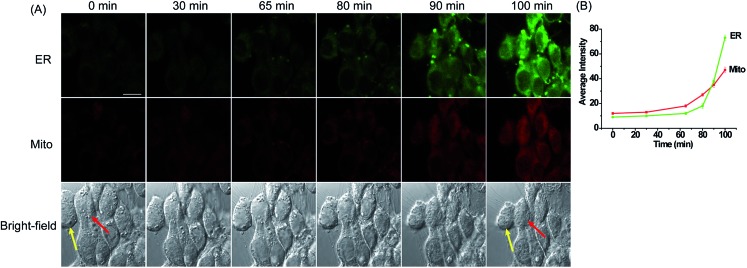
(A) Confocal fluorescence images of 4T1 cells stained simultaneously with **ER-H_2_O_2_** and **MI-H_2_O_2_** (10 μM) in the presence of BSO (5 mM) at different times. The first row (green channel) contains fluorescence images for **ER-H_2_O_2_** collected at 500–620 nm by excitation at 405 nm. The second row (red channel) contains fluorescence images for **MI-H_2_O_2_** collected at 550–600 nm by excitation at 543 nm. The third row contains bright-field images. (B) The output of average fluorescence intensity changes in image A at different times. Scale bar: 20 μm.

In an effort to explore the practicability of **ER-H_2_O_2_** and **MI-H_2_O_2_** for the simultaneous imaging of H_2_O_2_ in live cells, we first carried out fluorescence imaging of H_2_O_2_ in 4T1 cells treated with apoptotic stimulant *i.e.*l-buthionine sulfoximine (BSO). BSO is an inhibitor of gamma glutamyl cysteine synthetase (γGCS), which can induce a decline of glutathione (GSH) and increase of ROS within the whole cell that further results in cell apoptotic behavior.^51^ The 4T1 cells were simultaneously incubated with **ER-H_2_O_2_** and **MI-H_2_O_2_** for 30 min at 37 °C. Then, the cells were washed three times with PBS after the incubation medium was removed. BSO (5 mM) was added to induce apoptosis. As shown in Fig. 4, when the 4T1 cells were treated with BSO for various times, the fluorescence intensity of **ER-H_2_O_2_** (green channel) and **MI-H_2_O_2_** (red channel) gradually elevated simultaneously, implying the H_2_O_2_ concentration increased in both mitochondria and ER. At the same time, the shrinking morphologic changes of cells were observed, indicating the occurrence of apoptosis. A similar result was found in the HepG2 cells incubated with higher concentrations of BSO at different times (Fig. S15†). These results displayed that H_2_O_2_ levels both in the mitochondria and ER would rise during BSO induced apoptosis, presumably because attenuation of cellular GSH levels induced by BSO disturbed the cellular redox status.

**Fig. 5:**
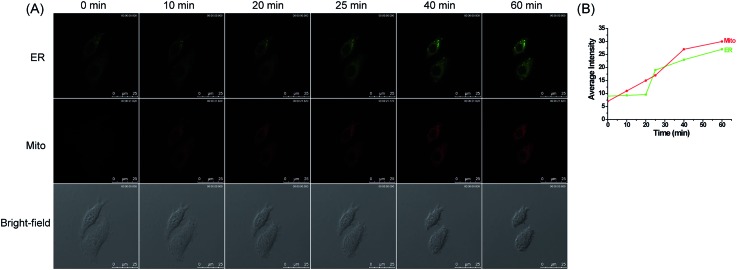
(A) Confocal fluorescence images of HepG2 cells stained simultaneously with **ER-H_2_O_2_** and **MI-H_2_O_2_** (10 μM) in the presence of CCCP (100 μM) at different times. The first row (green channel) contains fluorescence images for **ER-H_2_O_2_** collected at 500–620 nm by the excitation at 405 nm. The second row (red channel) contains fluorescence images for **MI-H_2_O_2_** collected at 550–600 nm by the excitation at 543 nm. The third row contains bright-field images. (B) The output of average fluorescence intensity changes in image (A) at different times. Scale bar: 25 μm.

Inspired by the above interesting results, we next intended to investigate the synergistic variations of H_2_O_2_ levels within these two organelles in cells treated with organelle-specific apoptotic stimuli. We first examined the changes on H_2_O_2_ levels in HepG2 cells treated with carbonyl cyanide *m*-chlorophenylhydrazone (CCCP). CCCP, an un-coupler of mitochondrial photophosphorylation, can induce apoptosis through a mitochondria-dependent pathway by disturbing the mitochondrial membrane potential, which can increase ROS generation in mitochondria.^52^,^53^ To explore the variations of H_2_O_2_ levels in the ER under this stimulant, the HepG2 cells were simultaneously incubated with **ER-H_2_O_2_** and **MI-H_2_O_2_** for 30 min at 37 °C. Then, the cells were washed three times with PBS after the incubation medium was removed. CCCP (100 μM) was added to induce mitochondria-oriented apoptosis. As indicated in Fig. 5, when the HepG2 cells were treated with CCCP for various times, the fluorescence intensity of **MI-H_2_O_2_** (red channel) gradually enhanced, indicating the elevation of H_2_O_2_ levels in the mitochondria under this stimulus. Whereas, the fluorescence intensity of **ER-H_2_O_2_** (green channel) was weak for the first 20 min, indicating no rise of H_2_O_2_ in the ER under CCCP induced apoptosis in the initial stage. However, the persistent stimulus resulted in a moderate fluorescence increase of **ER-H_2_O_2_**, suggesting a rise of H_2_O_2_ in the ER. In addition, the fluorescence imaging of HepG2 cells induced by rotenone,^54^,^55^ an inhibitor of mitochondrial respiratory chain complex I, produced similar results. H_2_O_2_ levels remained elevated in the mitochondria and a delayed rise in H_2_O_2_ levels occurred in the ER (Fig. S16†). All of this data showed that H_2_O_2_ levels prominently and continuously increased in mitochondria during mitochondria-oriented apoptotic stimulus, and sustaining apoptosis led to a subsequent H_2_O_2_ rise in the ER. The reason presumably results from the fact that dysfunction of mitochondria may elicit interplay with other organelles,^4^ such as the ER, the lysosome, and the nucleus, which may promote the coordinated variation of active molecules or inter-organellar cross-talk.

**Fig. 6:**
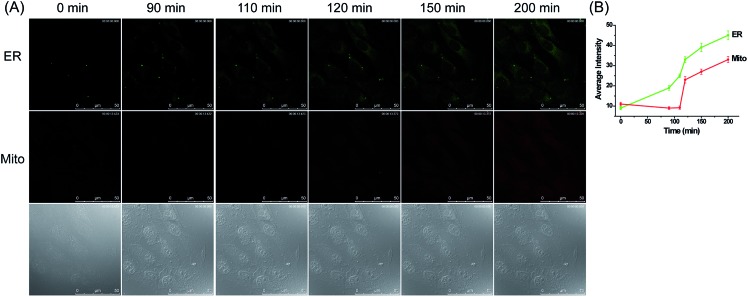
(A) Confocal fluorescence images of 4T1 cells stained simultaneously with **ER-H_2_O_2_** and **MI-H_2_O_2_** (10 μM) in the presence of Tm (100 μg mL^–1^) at different times. The first row (green channel) contains fluorescence images for **ER-H_2_O_2_** collected at 500–620 nm by the excitation at 405 nm. The second row (red channel) contains fluorescence images for **MI-H_2_O_2_** collected at 550–600 nm by the excitation at 543 nm. The third row contains bright-field images. (B) The output of average fluorescence intensity changes in image A at different times. Scale bar: 50 μm.

At last, **ER-H_2_O_2_** and **MI-H_2_O_2_** were utilized for simultaneous fluorescence imaging of H_2_O_2_ in 4T1 cells treated with Tm, an ER-oriented apoptotic stimulant. To explore whether the H_2_O_2_ levels would change in the mitochondria under this stimulant, the 4T1 cells were simultaneously incubated with **ER-H_2_O_2_** and **MI-H_2_O_2_** for 30 min at 37 °C. Then, cells were washed three times with PBS after the incubation medium was removed. Tm (100 μg mL^–1^) was added to induce ER-associated apoptosis. As illustrated in Fig. 6, when the 4T1 cells were treated with Tm for various times, the fluorescence intensity of **ER-H_2_O_2_** (green channel) gradually enhanced from about 90 min after addition of Tm, indicating the rise of H_2_O_2_ concentration in the ER under this stimulus. In the meantime, the fluorescence intensity of **MI-H_2_O_2_** was weak and almost stable within 120 min, and the fluorescence intensity increased progressively from 120 min to 200 min. All this data demonstrated that ER stress-derived apoptosis will upgrade H_2_O_2_ levels within the ER. Meanwhile, a delayed rise in H_2_O_2_ levels was observed in the mitochondria. We speculate that this phenomenon probably resulted from direct diffusion of H_2_O_2_ among different compartments^56^,^57^ or indirect generation in the mitochondria stimulated by calcium influx from the ER to the mitochondria during conditions of ER stress.^58^

## Conclusions

In conclusion, we have presented the application of two organelle-targeting fluorescent probes termed **MI-H_2_O_2_** and **ER-H_2_O_2_** for imaging H_2_O_2_ in the mitochondria and the ER of live cells during apoptosis with real-time operability and reliability. The data shows that **ER-H_2_O_2_** and **MI-H_2_O_2_** can selectively and sensitively detect exogenous and endogenous H_2_O_2_ under a myriad of stimuli. More importantly, **MI-H_2_O_2_** and **ER-H_2_O_2_** display distinct excitation and emission spectra, which favors multicolor fluorescence imaging in live cells. By utilizing **MI-H_2_O_2_** and **ER-H_2_O_2_** for simultaneous fluorescence imaging of H_2_O_2_ in live cells, we found that the variations in H_2_O_2_ levels in the mitochondria and the ER were different during apoptosis induced by various stimuli. H_2_O_2_ levels were enhanced in both the mitochondria and the ER during the BSO-treated apoptosis process. During mitochondria-related apoptosis, H_2_O_2_ levels are prominently and consistently increased in the mitochondria, and subsequently H_2_O_2_ elevation was found in the ER. Whereas, during ER-associated apoptosis, the ER is the major site for overproduction of H_2_O_2_, while a delayed rise in H_2_O_2_ levels was also observed in the mitochondria. Although precise H_2_O_2_ biology during apoptosis involving the mitochondria and the ER should be further studied, this was the first example of simultaneous fluorescence imaging of H_2_O_2_ in the mitochondria and the ER during apoptosis. This may offer a useful platform for further elucidating the molecular mechanism of apoptosis regulated by different organelles.

## Supplementary Material

Supplementary information
