# Supplementary material for: Simultaneous fluorescence imaging of hydrogen peroxide in mitochondria and endoplasmic reticulum during apoptosis
Source: Chem Sci. 2016 Jun 1;7(9):6153–9. doi: 10.1039/c6sc01793b (PMC6024174; doi:10.1039/c6sc01793b)
Supplement: Supplementary file 1 [file SC-007-C6SC01793B-s001.pdf]

Electronic Supplementary Material (ESI) for Chemical Science.
This journal is © The Royal Society of Chemistry 2016

Electronic Supplementary Information (ESI) for

Simultaneous fluorescence imaging of hydrogen peroxide in mitochondria and endoplasmic reticulum during apoptosis

Haibin Xiao, Ping Li,* Xiufen Hu, Xiaohui Shi, Wen Zhang, and Bo Tang*

College of Chemistry, Chemical Engineering and Materials Science, Collaborative Innovation Center of Functionalized Probes for Chemical Imaging in Universities of Shandong, Key Laboratory of Molecular and Nano Probes, Ministry of Education, Shandong Provincial Key Laboratory of Clean Production of Fine Chemicals, Shandong Normal University, Jinan 250014, P. R. China

E-mail: tangb@sdnu.edu.cn

Table of Contents

Materials and instruments
Cells culture
Determination of the limit of detection
Scheme S1 Synthesis of **MI-H_2O_2**
Scheme S2 Synthesis of **ER-H_2O_2**
Figure S1 The fluorescence intensity changes of **MI-H_2O_2** with different concentrations of H_2O_2
Figure S2 The fluorescence intensity ratio F_{558}/F_{458} of **ER-H_2O_2** with different concentrations of H_2O_2
Figure S3 The interference experiments of **MI-H_2O_2** with ROS and metal ions
Figure S4 The interference experiments of **ER-H_2O_2** with ROS and metal ions
Figure S5 The reaction kinetics for **MI-H_2O_2** and **ER-H_2O_2** with H_2O_2
Figure S6 Photostability experiments of **MI-H_2O_2** and **ER-H_2O_2** with H_2O_2
Figure S7 The confocal fluorescence imaging of **MI-H_2O_2** and **ER-H_2O_2** with commercial organelle dyes in 4T1 cells
Figure S8 The cytotoxicity assay of **MI-H_2O_2** and **ER-H_2O_2**
Figure S9 The fluorescence spectra of **ER-H_2O_2** and **MI-H_2O_2** with

H_2O_2 under different excitation wavelengths
Figure S10 The confocal fluorescence imaging of **MI-H_2O_2** and **ER-H_2O_2** in HepG2 cells stimulated with PMA or Tm
Figure S11 The confocal fluorescence imaging of **ER-H_2O_2** in 4T1 cells treated with Tm
Figure S12 The confocal fluorescence imaging of **ER-H_2O_2** in 4T1 cells treated with DTT
Figure S13 Confocal fluorescence images of HepG2 cells stained with **ER-H_2O_2** in the presence of nelfinavir
Figure S14 Confocal fluorescence images of 4T1 cells stained with **ER-H_2O_2** in the presence of nelfinavir
Figure S15 Confocal fluorescence images of HepG2 cells stained with **ER-H_2O_2** and **MI-H_2O_2** in BSO-induced apoptosis
Figure S16 Confocal fluorescence images of HepG2 cells stained with **ER-H_2O_2** and **MI-H_2O_2** during rotenone-induced apoptosis
References
The ^{1}HNMR, ^{13}CNMR, and HRMS original spectra

Materials and instruments

Unless otherwise stated, all reagents were purchased from commercial suppliers and used without further purification. The solvents were purified by conventional methods before use. ER-Tracker Red, and Mito-Tracker Deep Red were purchased from Invitrogen (USA). Phorbol 12-myristate 13-acetate (PMA), rotenone, carbonyl cyanide m-chlorophenylhydrazone (CCCP), lipopolysaccharide (LPS) and L-buthionine sulfoximine (BSO) were purchased from Sigma. Nelfinavir was purchased from Aladdin. Tunicamycin (Tm) was purchased from Solarbio Science and Technology. Silica gel (200-300 mesh) used for flash column chromatography was purchased from Qingdao Haiyang Chemical Co., Ltd. **MI-H_2O_2** and **ER-H_2O_2** were dissolved in dimethyl sulfoxide (DMSO) to produce 1 mM stock solutions. ^{1}HNMR and ^{13}CNMR spectra were determined by 300 MHz and 75 MHz using Bruker NMR spectrometers. The mass spectra were obtained by Bruker maxis ultra-high resolution-TOF MS system. The fluorescence spectra measurements were performed using FLS-920 Edinburgh fluorescence spectrometer. Cary eclipse fluorescence spectrophotometer was used for the kinetic assays. Fluorescence imaging in cells were performed with Leica TCS SP5 Confocal Laser Scanning Microscope. The laser power of confocal imaging is 5 mW (405 nm laser) and 15 mW (514, 543 and 633 nm laser). The mouse mammary carcinoma 4T1 cells and human

hepatoma cells (HepG2) were purchased from Cell Bank of the Chinese Academy of Sciences (Shanghai, China).

Cells culture

Human hepatoma (HepG2) and mouse mammary carcinoma (4T1) cells were cultured in high glucose DMEM (4.5 g of glucose/L) or RPMI 1640 supplemented with 10% fetal bovine serum, 1% penicillin, and 1% streptomycin at 37 °C in a 5% CO_2 /95% air incubator MCO-15AC (SANYO, Tokyo, Japan). One day before imaging, the cells were detached and were replanted on glass-bottomed dishes.

Determination of the limit of detection

The detection limit was determined from the fluorescence titration data. So the detection limit was calculated with the following equation: Detection limit=3σ/k, where σ is the standard deviation of blank measurement, k is the slop between the fluorescence intensity or fluorescence intensity ratio versus H_2O_2 concentration.

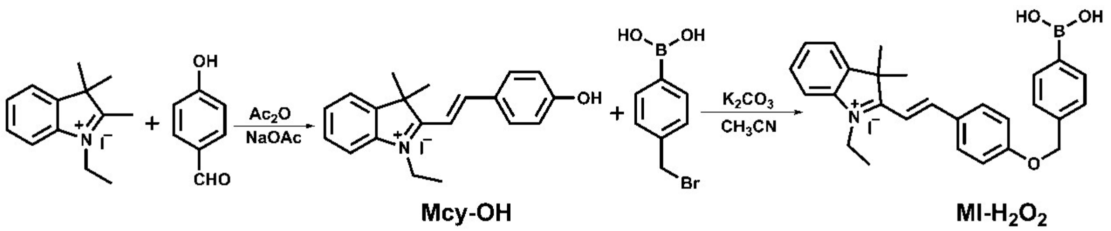

Scheme S1 Synthesis of **MI-H_2O_2**

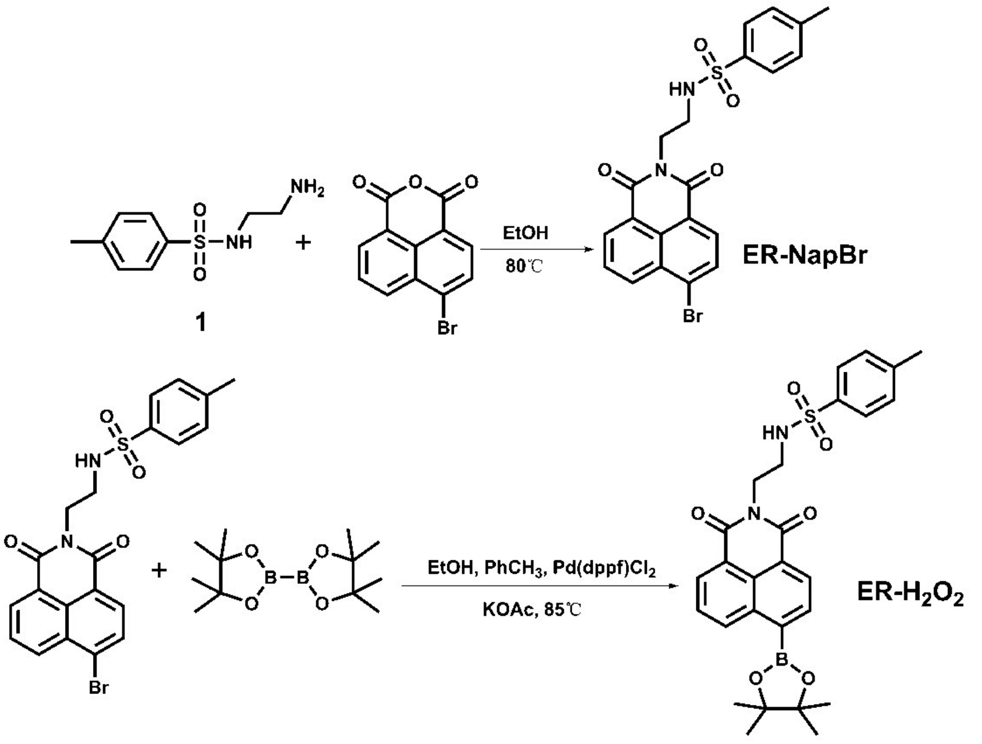

Scheme S2 Synthesis of **ER-H_2O_2**

Synthesis of compound **Mcy-OH** and probe **MI-H_2O_2**

Compound **Mcy-OH**: Under the Ar gas condition, 2,3,3-trimethyl-1-ethyl-3H-indolium iodide[1] (0.315 g, 1 mmol) and 4-hydroxybenzaldehyde (146.5 mg, 1.2 mmol), sodium acetate (0.25 g, 3.0 mmol) were dissolved in 10 mL of acetic anhydride. The mixture was stirred at 55℃ for 2 h. Then the solvents were dropped in ethyl ether, and the residue was purified by silica gel chromatography with dichloromethane/methanol (10:1 v/v) to give **MCy-OH** as a red solid (0.27 g, 64 %). ^{1}H NMR (300 MHz, *d6*-DMSO) δ(ppm): 1.430 (s, 3H), 1.784 (s, 6H), 4.655 (s, 2H), 6.949 (s, 2H), 7.428 (d, *J*=10.8 Hz, 1H), 7.589 (s, 2H), 7.861 (s, 2H), 8.166 (s, 2H), 8.383 (d, *J*=10.8 Hz, 1H). ^{13}C NMR (75 MHz, *d6*-DMSO) δ(ppm): 14.0, 26.5, 41.9, 52.2, 108.6, 114.9,

117.2, 123.5, 126.3, 129.1, 129.5, 134.5, 141.0, 144.0, 154.9, 164.7, 181.1. HRMS (ESI) m/z calcd. for $C_{20}H_{22}NO^+$ 292.1696, found 292.1646.

Probe **MI-H_2O_2**: Compound **MCy-OH** (0.21 g, 0.5 mmol) and 4-(bromomethyl)phenylboronic acid (0.10 g, 0.5 mmol), potassium carbonate (0.138 g, 1.0 mmol) were dissolved in 15 mL acetonitrile. The mixture was stirred at 80℃ for 6 h under the Ar gas condition. The residue was purified by silica gel chromatography with dichloromethane /methanol (20:1 v/v) to give **MI-H_2O_2** as a light yellow solid (0.12 g, 43 %). ^1H NMR (300 MHz, *d6*-DMSO) δ(ppm): 1.426 (t, *J*=6.9 Hz, 3H), 1.774 (s, 6H), 4.657 (q, *J*=6.9 Hz, 2H), 5.276 (s, 2H), 7.248 (d, *J*=9.0 Hz, 2H), 7.441 (d, *J*=7.8 Hz, 2H), 7.586 (m, 3H), 7.822 (d, *J*=7.8 Hz, 2H), 7.870 (m, 2H), 8.052 (s, 2H), 8.250 (d, *J*=9.0 Hz, 2H), 8.455 (d, *J*=16.2 Hz, 1H). ^{13}C NMR (75 MHz, *d6*-DMSO) δ(ppm): 14.1, 26.3, 42.3, 52.5, 70.3, 110.3, 115.3, 116.2, 123.6, 127.2, 128.0, 129.6, 133.8, 134.8, 138.5, 140.9, 144.2, 154.5, 163.5, 181.7. HRMS (ESI) m/z calcd. for $C_{27}H_{29}BNO_3^+$ 426.2239, found 426.2315.

Synthesis of **ER-NapBr** and probe **ER-H_2O_2**

Compound **ER-NapBr**: Under the Ar gas condition, compound **1**[2] (0.43 g, 2 mmol) and 4-bromo-1,8-naphthalic anhydride (0.56 g, 2 mmol) were dissolved in 20 mL ethanol. The reaction was heated to 80℃ for 8 h. The mixture was poured into 40 mL distilled deionized water, and the solid was filtered and purified by column chromatography with CH_2Cl_2:

CH_3OH=20:1 as the eluent. Compound **ER-NapBr** (0.38 g, 40% yield) was obtained as a grey white solid. ^1H NMR (300 MHz, *d6*-DMSO) δ(ppm): 2.235 (s, 3H), 3.096 (s, 2H), 4.086 (s, 2H), 7.200 (s, 2H), 7.563 (s, 2H), 7.766 (s, 1H), 7.953 (s, 1H), 8.226 (d, *J*=15.9 Hz, 2H), 8.479 (s, 2H). ^{13}C NMR (75 MHz, *d6*-DMSO) δ(ppm): 19.0, 21.3, 56.5, 122.5, 123.3, 126.8, 128.8, 129.2, 129.5, 129.9, 130.2, 131.3, 131.7, 131.9, 132.9, 138.2, 142.9, 163.3, 163.4. HRMS (ESI) m/z calcd. for $C_{21}H_{17}BrN_2O_4S$ [M+Na^+]: 494.9985, 496.9965, found 494.9982, 496.9959

Probe **ER-H_2O_2**: In an atmosphere of argon, to a mixture of compound **ER-NapBr** (0.24 mg, 0.5 mmol, 1.0 equiv.) in ethanol and toluene (v/v 1:1, 20 mL) was added Pd(dppf)Cl_2 (36.4 mg, 0.05 mmol, 0.1 equiv.), potassium acetate (147 mg, 1.5 mmol, 3.0 equiv.), and bis(pinacolato)-diboron (356 mg, 1.0 mmol, 2.0 equiv.). The reaction mixture was stirred for 10 h at 85℃ in an oil bath. H_2O (20 mL) was added and the mixture was extracted with 20 mL of CH_2Cl_2 thrice. The combined extracts were dried over Na_2SO_4. Concentration of the product and chromatography were carried out over silica gel (50:1 CH_2Cl_2:CH_3OH). This resulted in 91 mg (yield 35%) of **ER-H_2O_2** as a light yellow solid. ^1H NMR (300 MHz, $CDCl_3$) δ (ppm): 1.466 (s, 12H), 1.859 (s, 3H), 3.493 (q, *J*=5.1 Hz, 2H), 4.272 (t, *J*=5.1 Hz, 2H), 5.167 (t, *J*=4.8 Hz, 1H), 6.658 (d, *J*=8.1 Hz, 2H), 7.506 (d, *J*=8.1 Hz, 2H), 7.772 (m, 1H), 8.312 (d, *J*=7.2 Hz, 1H),

8.473 (m, 2H), 9.156 (d, *J*=8.4 Hz, 1H). ^{13}C NMR (75 MHz, $CDCl_3$) δ(ppm): 21.1, 24.9, 39.1, 42.5, 84.7, 122.0, 124.0, 126.6, 127.1, 127.7, 129.1, 129.9, 131.1, 134.3, 135.1, 135.3, 135.7, 137.0, 142.6, 164.6，164.7. HRMS (ESI) m/z calcd. for $C_{27}H_{29}BN_2O_6S$ [M+Na^+]: 543.1736, found 543.1727.

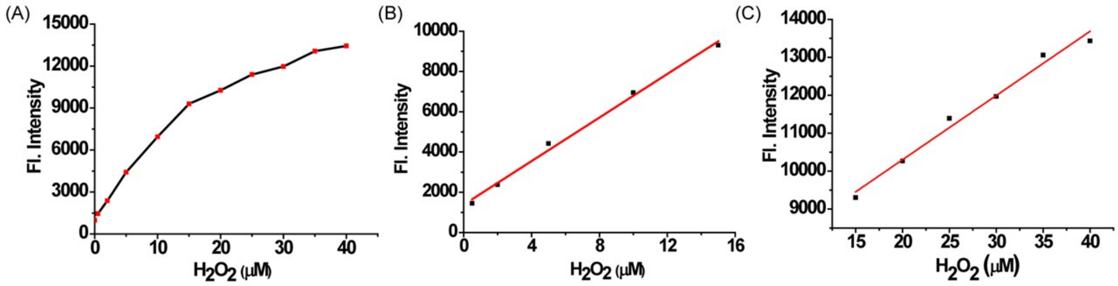

Figure S1 (A) The fluorescence intensity changes of **MI-H_2O_2** with different concentrations of H_2O_2 (0-40 μM). (B) and (C) were two linear regressions between fluorescence intensity and H_2O_2 concentration. There is a good linear correlation in the range of 0.5-15 μM H_2O_2 (y=553.14x+1249.42, R^2=0.9925) and 15-40 μM H_2O_2 (y=169.23x+6915.80, R^2=0.9805).

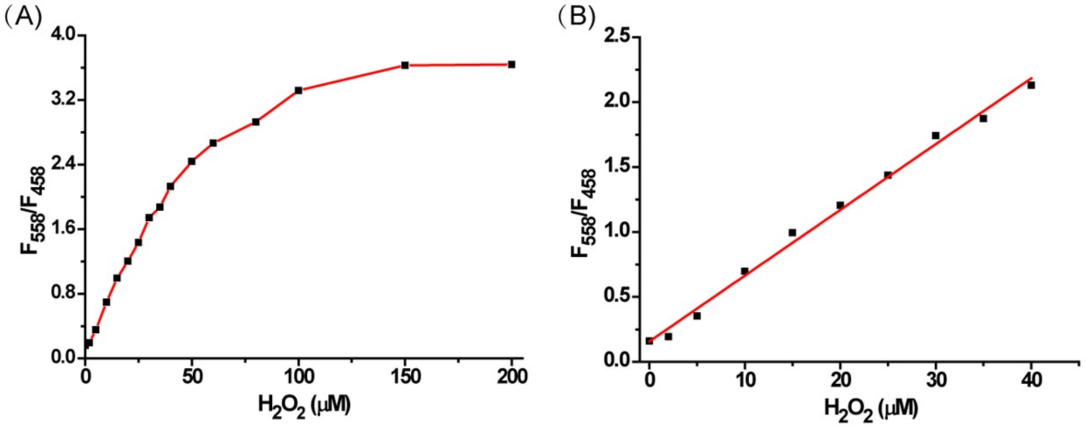

Figure S2 (A) The fluorescence intensity ratio F_{558}/F_{458} of **ER-H_2O_2** with different concentrations of H_2O_2 (0-200 μM). (B) The good linearity between ratio F_{558}/F_{458} and H_2O_2 concentration in the range of 0-40 μM.

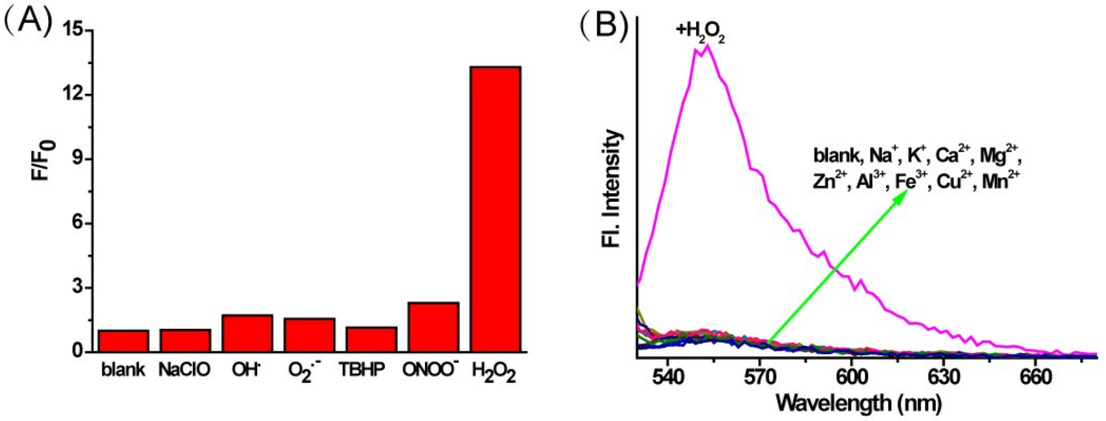

Figure S3 (A) The fluorescence responses of **MI-H_2O_2** with various ROS (H_2O_2: 50 μM, other ROS: 100 μM). (B) The fluorescence spectra of **MI-H_2O_2** with different metal ions (Na^+, K^+: 5 mM; Ca^{2+}, Mg^{2+}: 500 μM; Zn^{2+}, Al^{3+}, Fe^{3+}, Cu^{2+}, Mn^{2+}: 200 μM; H_2O_2: 50 μM). E_x=525 nm.

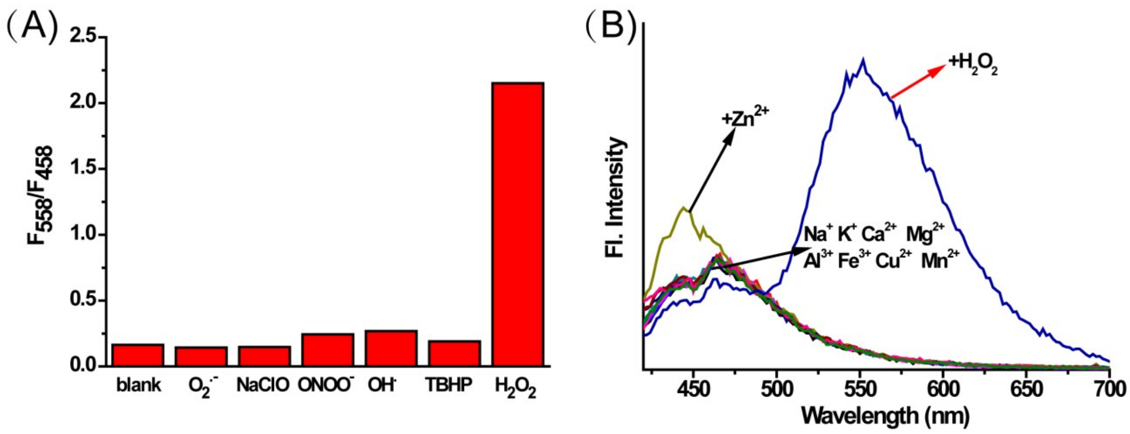

Figure S4 (A) The fluorescence intensity ratio F_{558}/F_{458} of **ER-H_2O_2** with various ROS (H_2O_2: 50 μM, other ROS: 100 μM). (B) The fluorescence spectra of **ER-H_2O_2** with different metal ions (Na^+, K^+: 5 mM; Ca^{2+}, Mg^{2+}: 500 μM; Zn^{2+}, Al^{3+}, Fe^{3+}, Cu^{2+}, Mn^{2+}: 200 μM; H_2O_2: 50 μM). E_x=400 nm.

Kinetic Studies:

The kinetic profiles of the reaction were performed under *pseudo*-first-order conditions with a large excess of H_2O_2 (100 equiv.) over probes **MI-H_2O_2** and **ER-H_2O_2** at room temperature. The *pseudo*-first-order rate constant *k'* for the reaction was calculated to be 4.35×10^{-3} S^{-1} and 2.35×10^{-3} S^{-1} for **MI-H_2O_2** and **ER-H_2O_2** respectively according to eqn (1):

$$\ln [(Fmax - Ft)/Fmax] = -k't \qquad (1)$$

Where Ft and Fmax are the fluorescence intensities at time t and the maximum value obtained after the reaction was complete (555 nm for **MI-H_2O_2** and 558 nm for **ER-H_2O_2**). *k'* is the pseudo-first-order rate

constant.

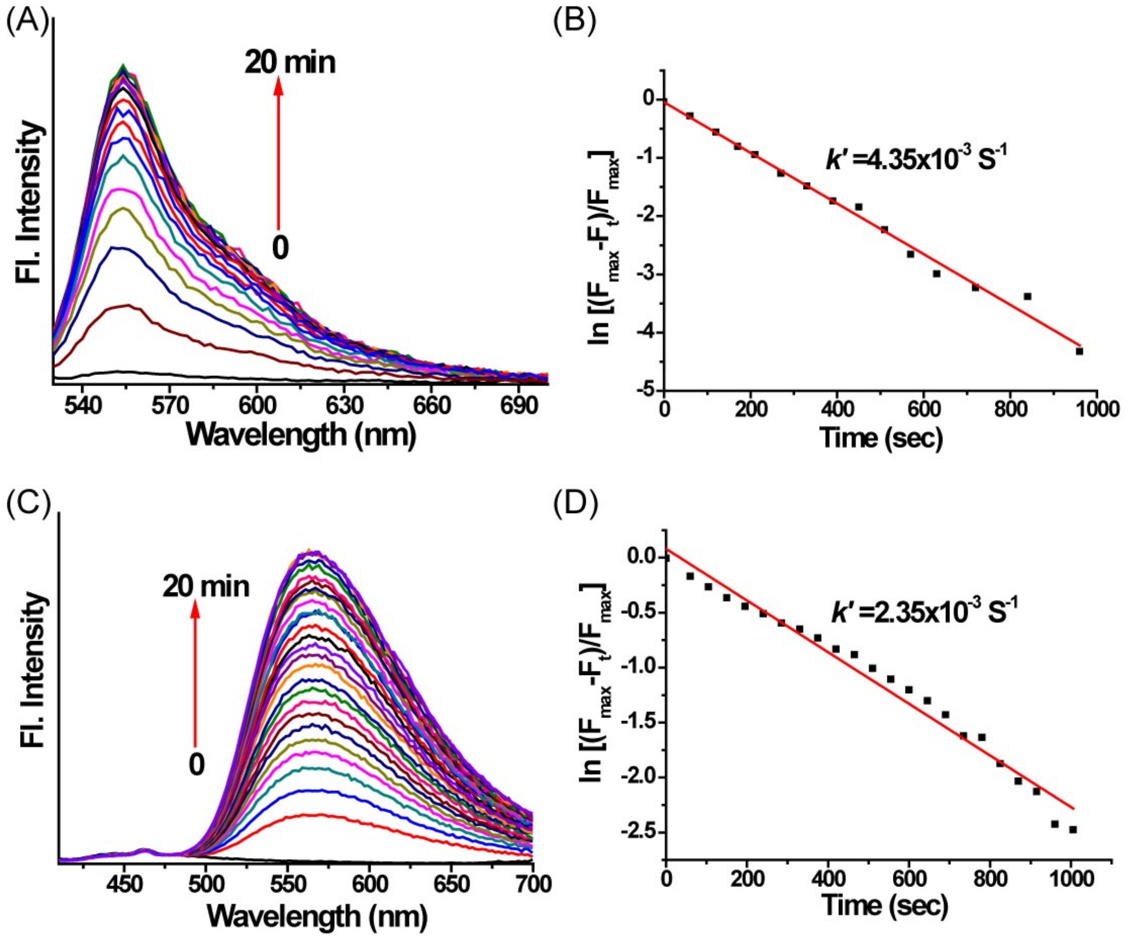

Figure S5 Fluorescence responses of 10 μM **MI-H_2O_2** (A, B) and **ER-H_2O_2** (C, D) to 1 mM H_2O_2 with excitation at 525 nm and 400 nm, respectively. The k'= 4.35 × 10^{-3} and 2.35 × 10^{-3} s^{-1} for **MI-H_2O_2** and **ER-H_2O_2** were obtained from slope of the plot of ln [(Fmax–Ft)/Fmax] to time (measured at 555 nm for **MI-H_2O_2** and 558 nm for **ER-H_2O_2**).

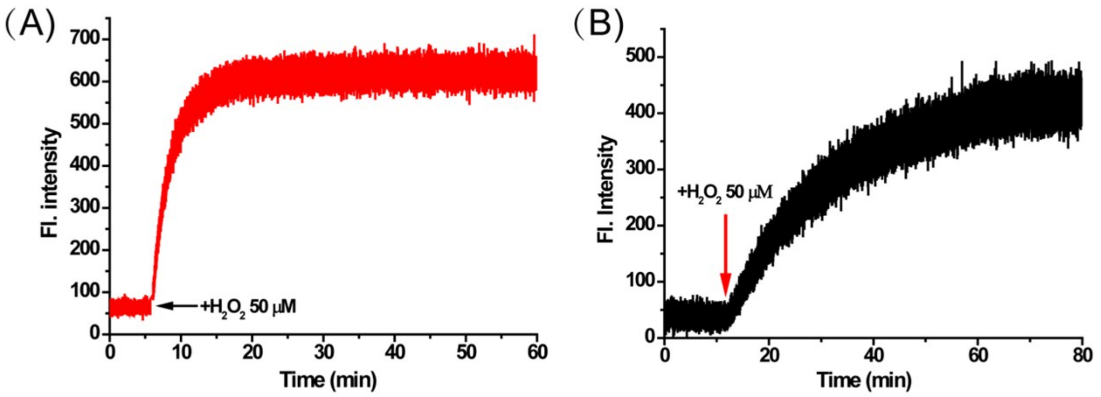

Figure S6 The photostability experiments of **MI-H_2O_2** and **ER-H_2O_2** with H_2O_2. (A) The changes for fluorescence intensity of **MI-H_2O_2** at 555 nm when excited with 525 nm. (B) The changes for fluorescence intensity of **ER-H_2O_2** at 558 nm when excited with 400 nm. Cary eclipse fluorescence spectrophotometer was used for these assays.

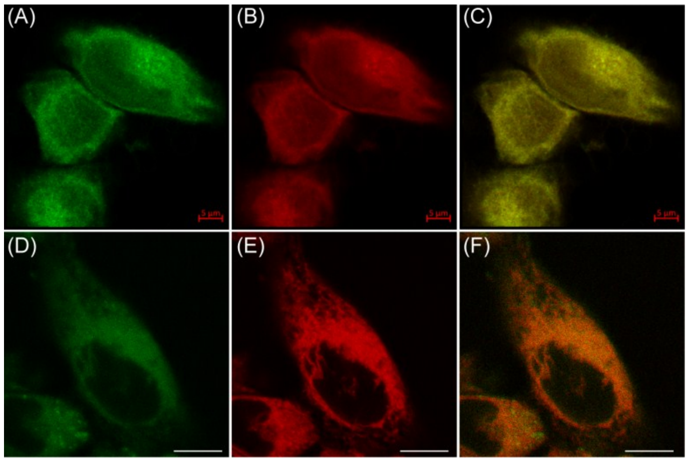

Figure S7 Confocal fluorescence images of 4T1 cells stained with **MI-H_2O_2** (A-C) and **ER-H_2O_2** (D-F) with commercial organelle dyes. (A) Fluorescence image of **MI-H_2O_2** (10 μM) in cells treated with 200 μM H_2O_2 for 60 min (green channel, Ex=514 nm, collected 520-580 nm) and (B) 0.5 μM Mito-Tracker Deep Red (red channel, Ex=633 nm, collected 640-700 nm). (C) Overlay of (A) and (B). Scale bar: 5 μm. (D)

Fluorescence image of **ER-H_2O_2** (10 μM) in cells treated with 200 μM H_2O_2 for 60 min (green channel, Ex=405 nm, collected 500-620 nm) and (E) 0.5 μM ER-Tracker Red (red channel, Ex=543 nm, collected 580-630 nm). (F) Overlay of (D) and (E). Scale bar: 10 μm.

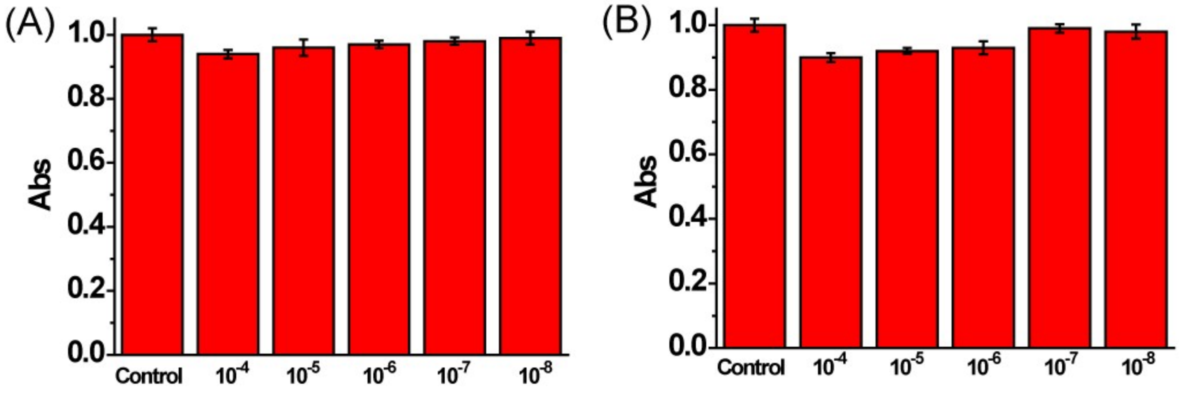

Figure S8 The MTT assays of **MI-H_2O_2** (A) and **ER-H_2O_2** (B) at different concentrations.

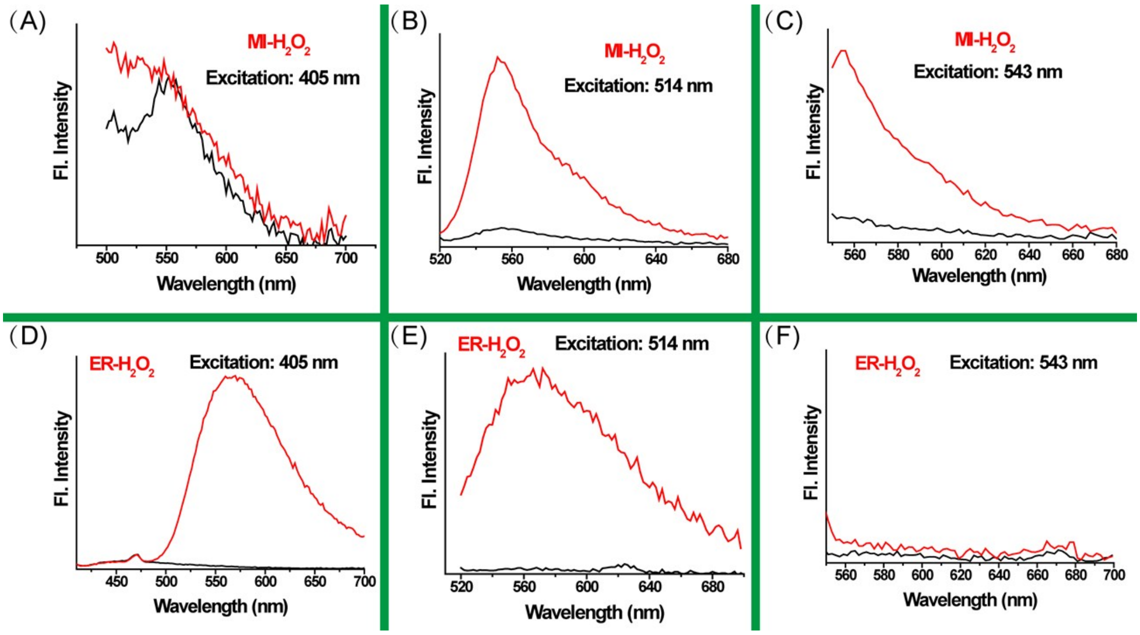

Figure S9 The fluorescence spectra of **ER-H_2O_2** and **MI-H_2O_2** with

H_2O_2 under different excitation wavelengths (405, 514 543 nm). (A-C) The fluorescence spectra of **MI-H_2O_2** with H_2O_2 under excitation of 405 nm (A), 514 nm (B) and 543 nm (C), respectively. (D-F) The fluorescence spectra of **ER-H_2O_2** with H_2O_2 under excitation of 405 nm (D), 514 nm (E) and 543 nm (F), respectively. The fluorescence enhancements of the two probes can be easily excited by 514 nm, which will lead the intense spectral overlap for confocal fluorescence imaging. To completely avoid the spectral overlap for confocal fluorescence imaging, we chose 543 nm as excitation wavelength for **MI-H_2O_2**. It is because **ER-H_2O_2** can't be excited at 543 nm, but can be easily excited by 405 nm. In stark contrast, fluorescence enhancement of **MI-H_2O_2** to H_2O_2 can be readily excited by 543 nm but not 405 nm.

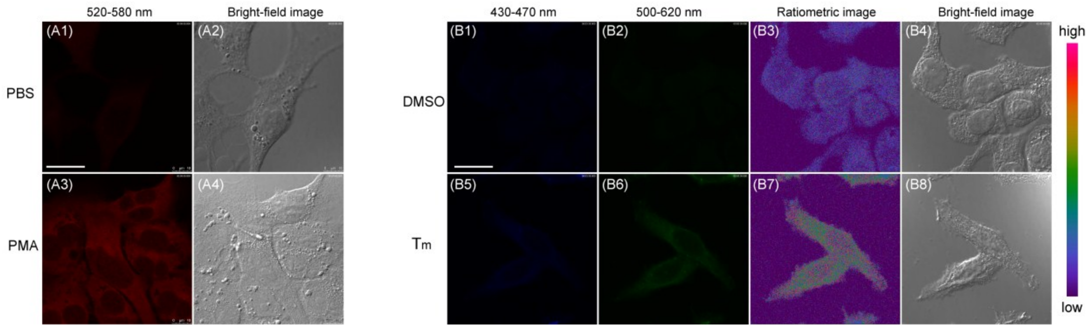

Figure S10 Confocal fluorescence images of live HepG2 cells stained with **MI-H_2O_2** and **ER-H_2O_2** under stimulus. (A1-A2) Cells incubated with PBS for 1 h and then stained with **MI-H_2O_2** (10 μM) for 1 h. (A3-A4) Cells incubated with PMA (100 μg/mL) for 1 h and then stained with **MI-H_2O_2** (10 μM) for 1 h. Ex=514 nm, collected 520-580 nm. Scare bar:

20 μm. (B1-B4) Cells incubated with DMSO (1%) for 8 h and then stained with **ER-H_2O_2** for 1 h. (B5-B8) Cells incubated with Tm (20 μg/mL) for 8 h and then stained with **ER-H_2O_2** for 1 h. Ex=405 nm, collected 430-470 nm for blue channel, 500-620 nm for green channel. Scare bar: 20 μm.

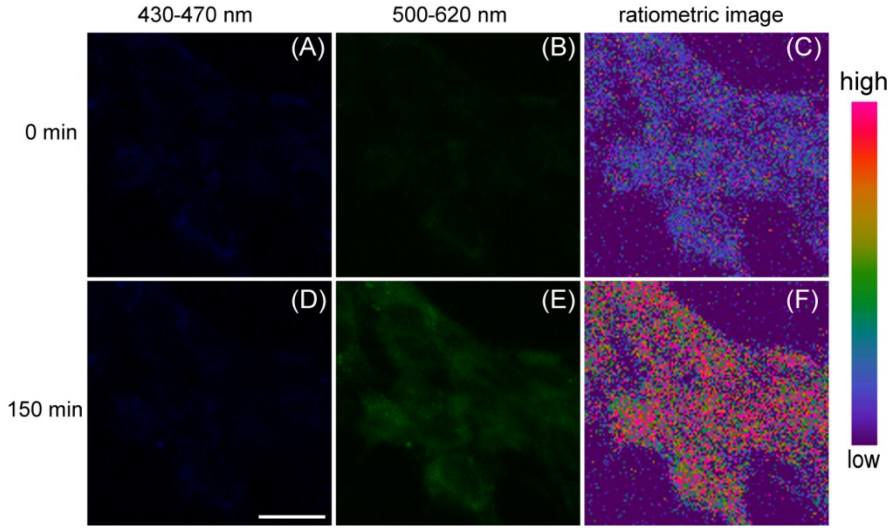

Figure S11 The ratiometric fluorescence imaging of **ER-H_2O_2** in 4T1 cells treated with Tm. 4T1 cells were incubated with 10 μM **ER-H_2O_2** for 1 h, and then were treated with 25 μg/mL Tm over a period of 2.5 h. Obviously, the fluorescence intensity in green channel (500-620 nm) at 150 min was higher than that at 0 min. And the ratio between green to blue channel was increased with time. Ex=405 nm. Scare bar: 20 μm.

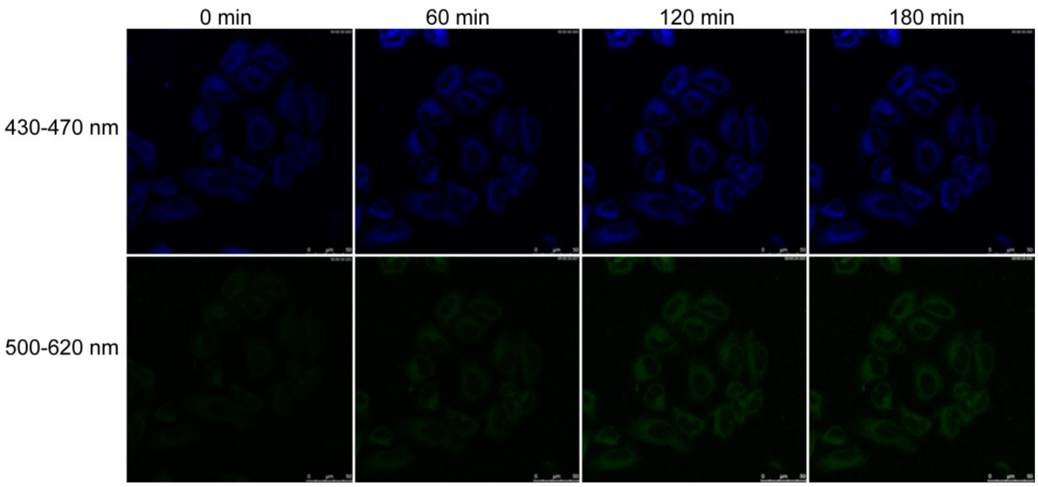

Figure S12 The confocal fluorescence imaging of **ER-H_2O_2** (10 μM) with addition of 10 mM DTT. The green channel displayed distinguishable and increasing fluorescence intensity over time. Ex=405 nm. Scare bar: 50 μm

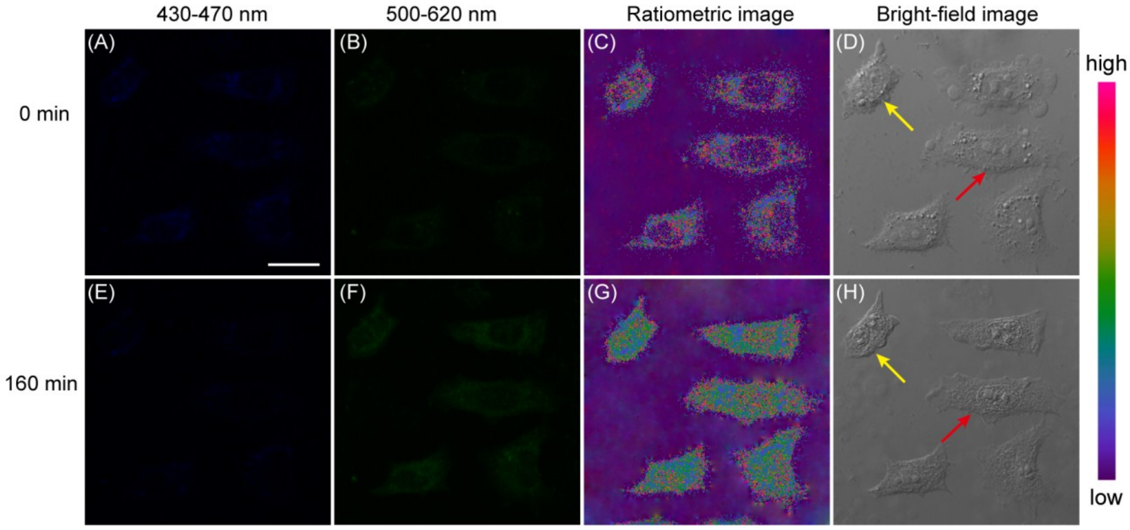

Figure 13 Confocal fluorescence images of live HepG2 cells stained with **ER-H_2O_2** (10 μM) in the presence of nelfinavir (200 μg/mL) at 0 and 160 min. (A) and (E) were blue channel collected 430-470 nm. (B) and (F) were green channel collected 500-620 nm. (C) and (G) were ratiometric

images between green and blue channel. (D) and (H) were bright-field images. The yellow and red arrows indicated the striking morphologic changes of two cells. Ex=405 nm. Scale bar: 20 μm.

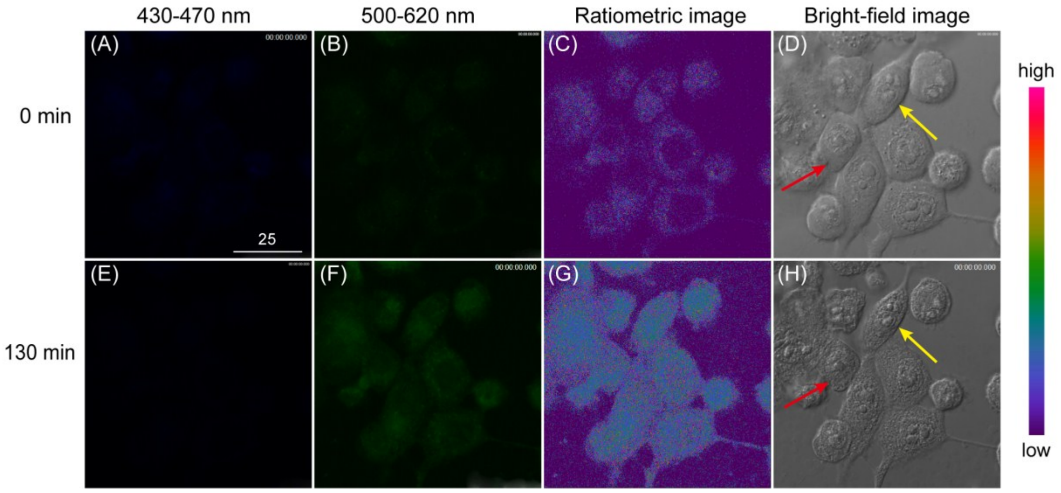

Figure S14 Confocal fluorescence images of 4T1 cells stained with **ER-H_2O_2** (10 μM) in the presence of nelfinavir (200 μg/mL) at 0 and 130 min. (A) and (E) were blue channel collected 430-470 nm. (B) and (F) were green channel collected 500-620 nm. (C) and (G) were ratiometric images between green and blue channel. (D) and (H) were bright-field images. The yellow and red arrows indicated the striking morphologic changes of two cells. Ex=405 nm. Scale bar: 25 μm.

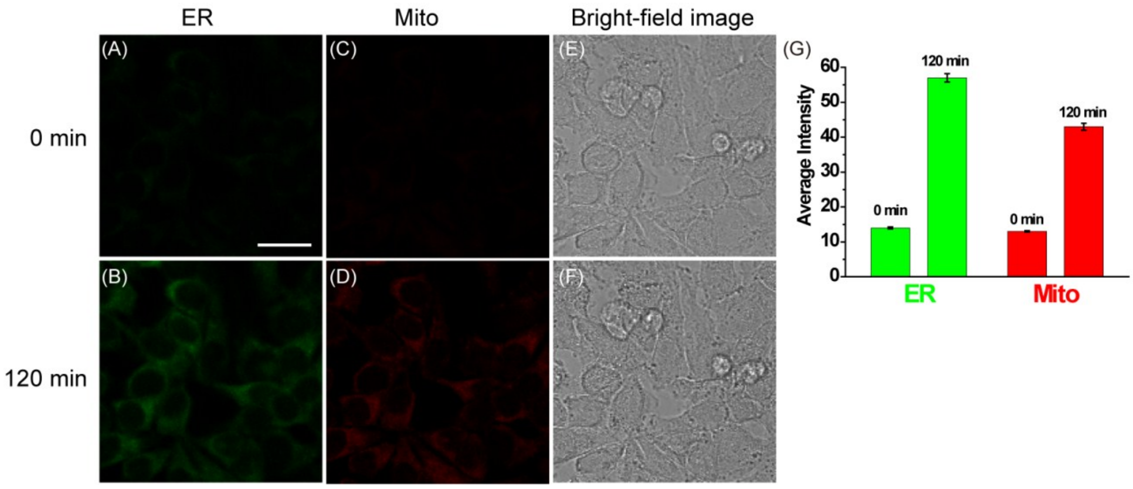

Figure S15 (A) Confocal fluorescence images of HepG2 cells stained with **ER- H_2O_2** and **MI-H_2O_2** (10 μM) in the presence of BSO (10 mM) at different time. (A-B) Fluorescence image of **ER-H_2O_2** collected 500-620 nm (Ex=405 nm). (C-D) Fluorescence image of **MI-H_2O_2** collected 550-600 nm (Ex=543 nm). (E-F) The bright-field images. (G) The output of average intensity in A-D. Scale bar: 25 μm.

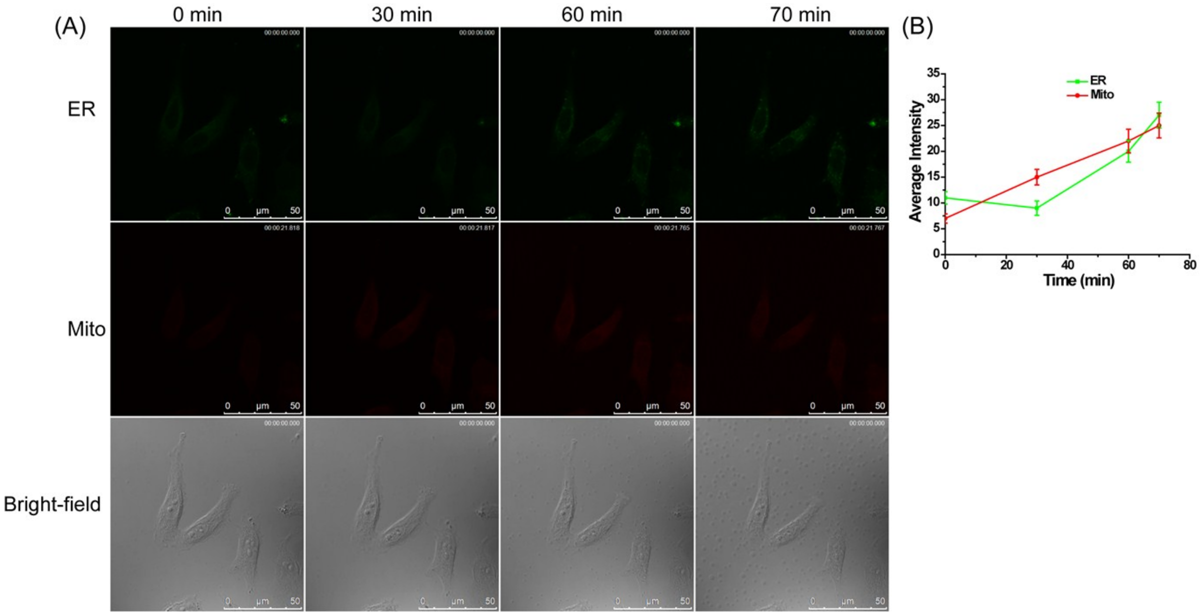

Figure S16 (A) Confocal fluorescence images of HepG2 cells stained

with **ER-H_2O_2** and **MI-H_2O_2** (10 μM) in the presence of rotenone (100 μM) at different time. The first row (green channel) was fluorescence images for **ER-H_2O_2** collected 500-620 nm by the excitation at 405 nm. The second row (red channel) was fluorescence images for **MI-H_2O_2** collected 550-600 nm by the excitation at 543 nm. The third row was bright-field images. (B) The output of average fluorescence intensity changes in image A at different time. Scale bar: 50 μm.

References

[1] F. Kong, R. Liu, R. Chu, X. Wang, K. Xu, and B. Tang, *Chem. Commun.,* 2013, **49**, 9176– 9178.

[2] M. T. Barros, and F. Siñeriz, *Tetrahedron,* 2000, **56**, 4759-4764.

The ^{1}HNMR, ^{13}CNMR, and HR-MS original spectra

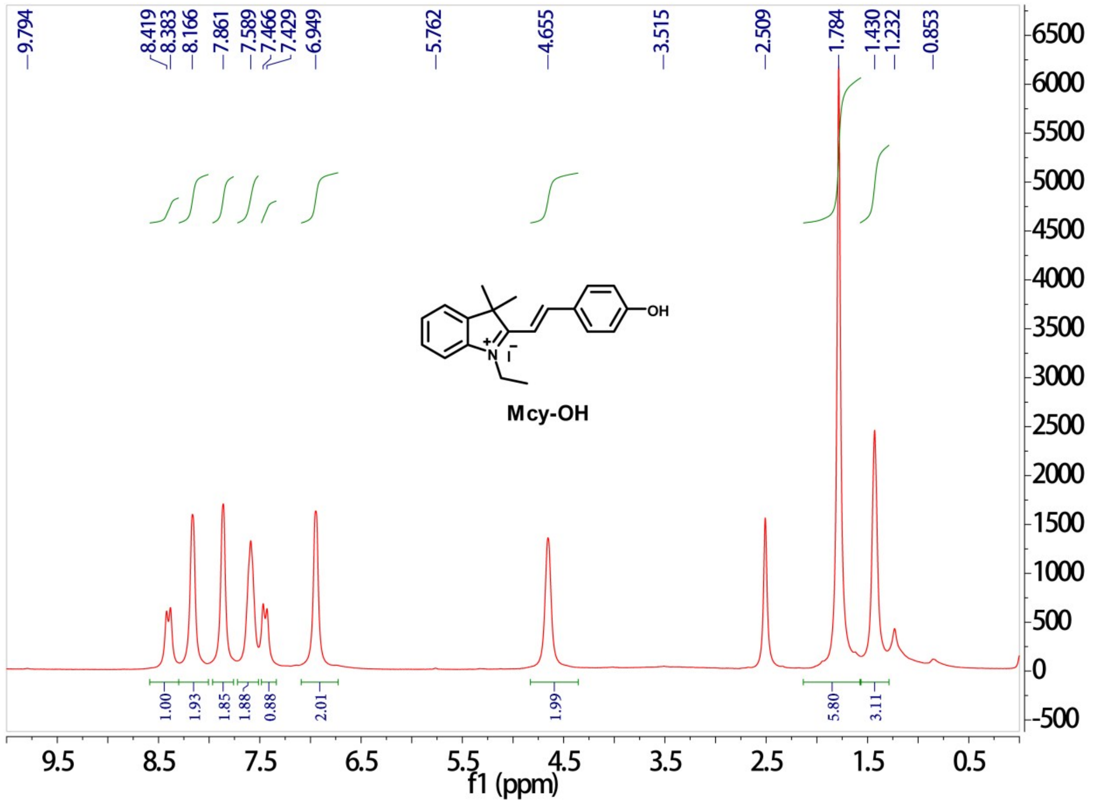

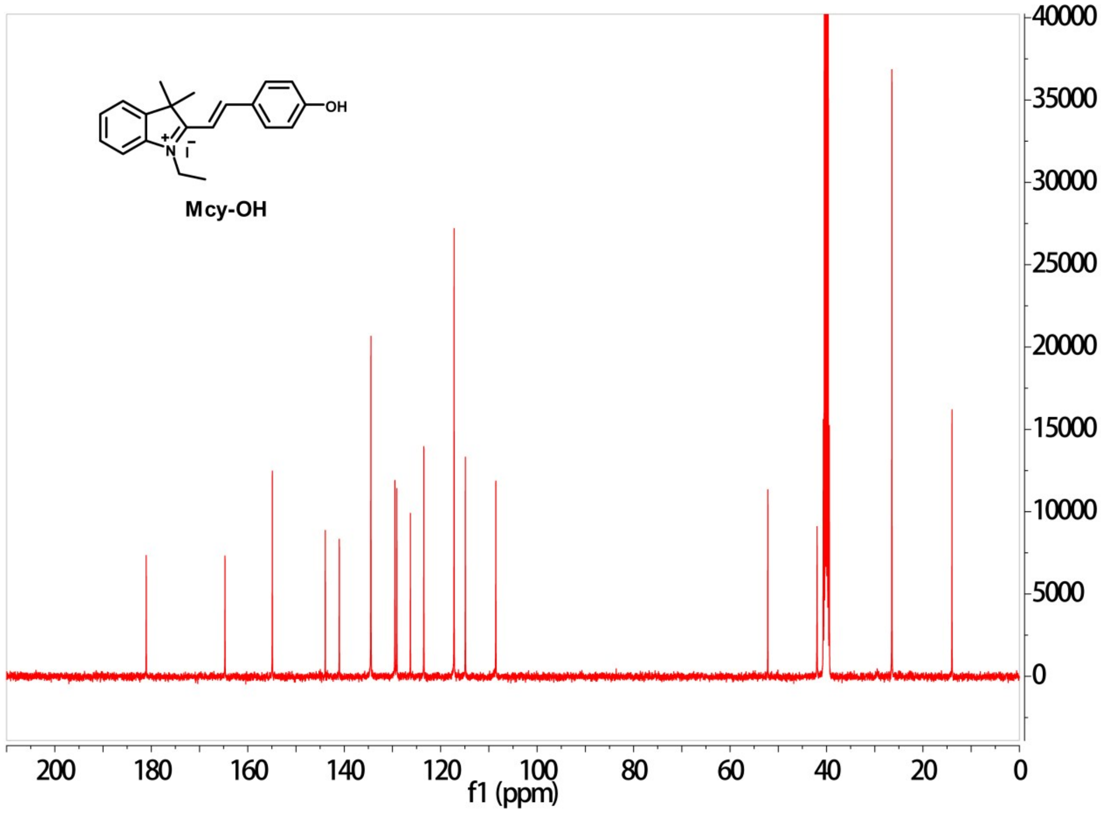

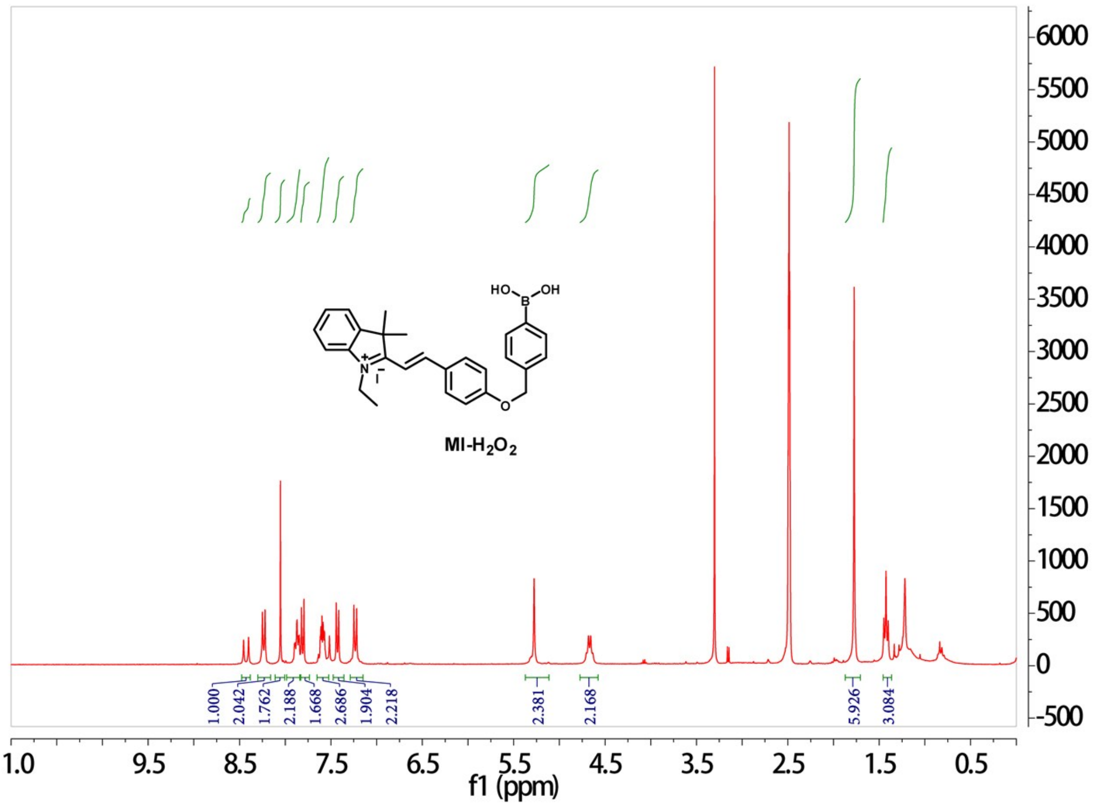

MI-H$_2$O$_2$
1.000
2.042
1.762
2.188
1.668
2.686
1.904
2.218
2.381
2.168
5.926
3.084
f1 (ppm)

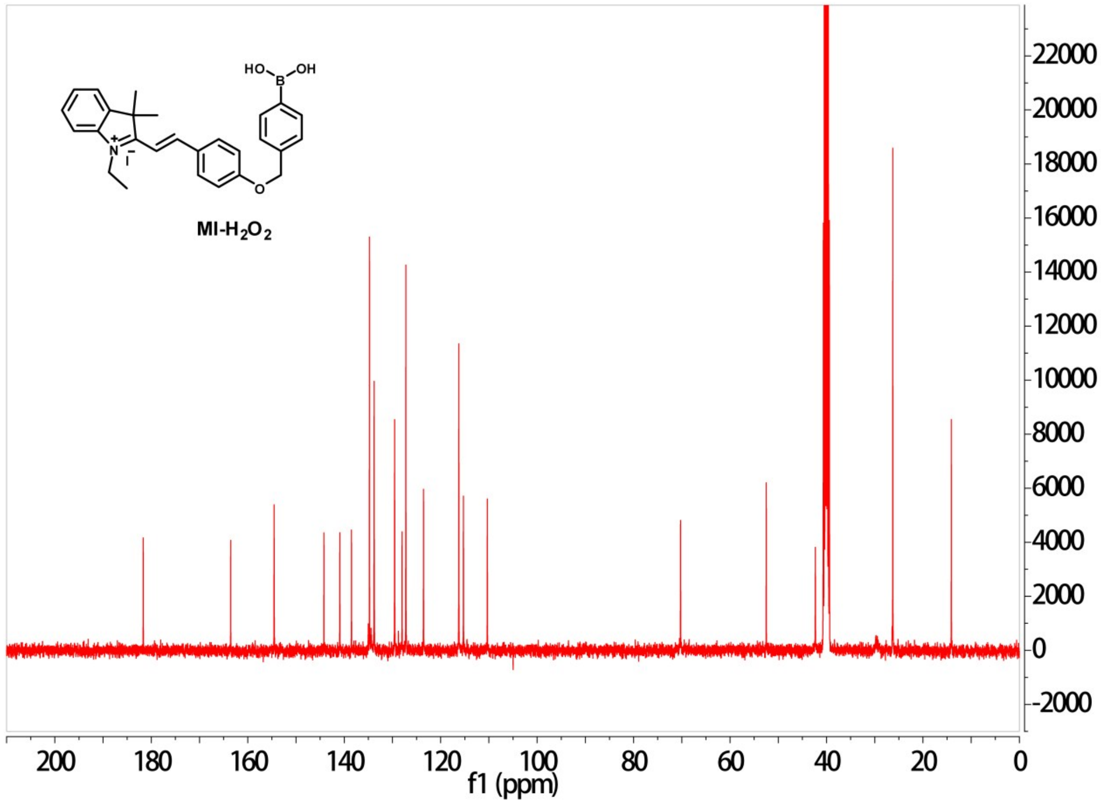

MI-H$_2$O$_2$
f1 (ppm)

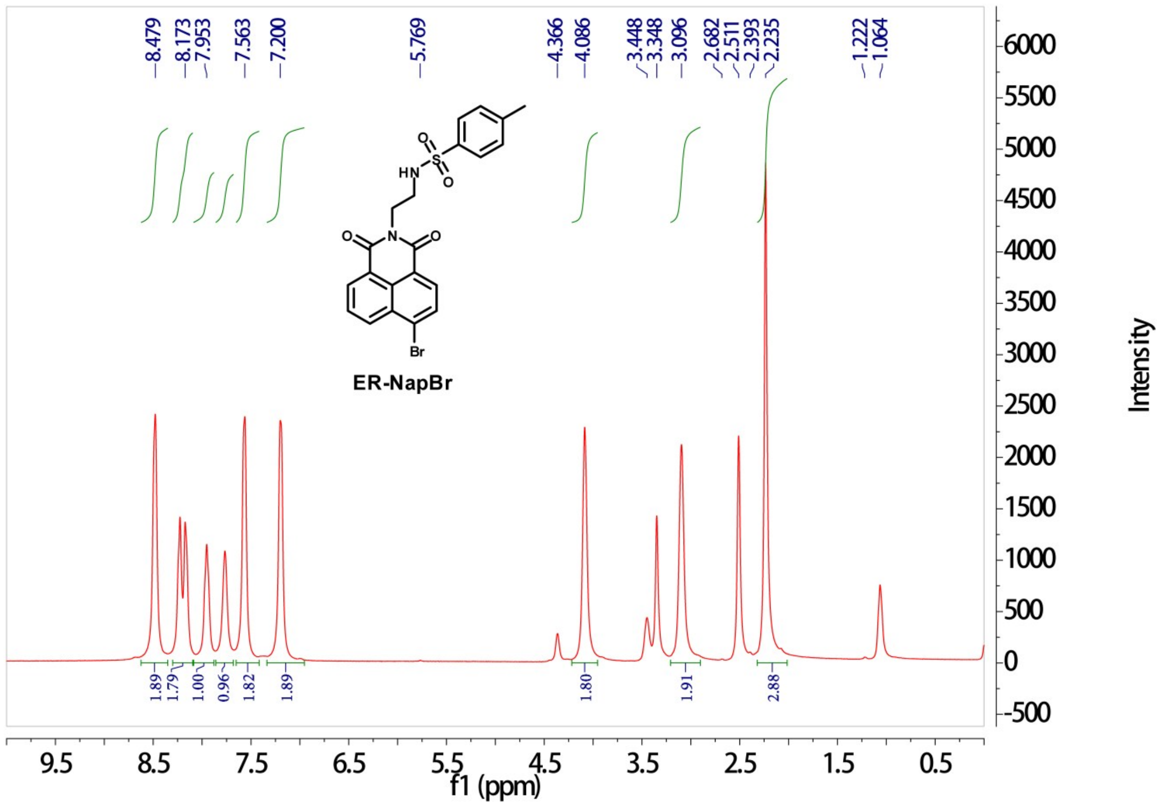
8.479
8.173
7.953
7.563
7.200
5.769
4.366
4.086
3.448
3.348
3.096
2.682
2.511
2.393
2.235
1.222
1.064
ER-NapBr
1.89
1.79
1.00
0.96
1.82
1.89
1.80
1.91
2.88
f1 (ppm)
Intensity

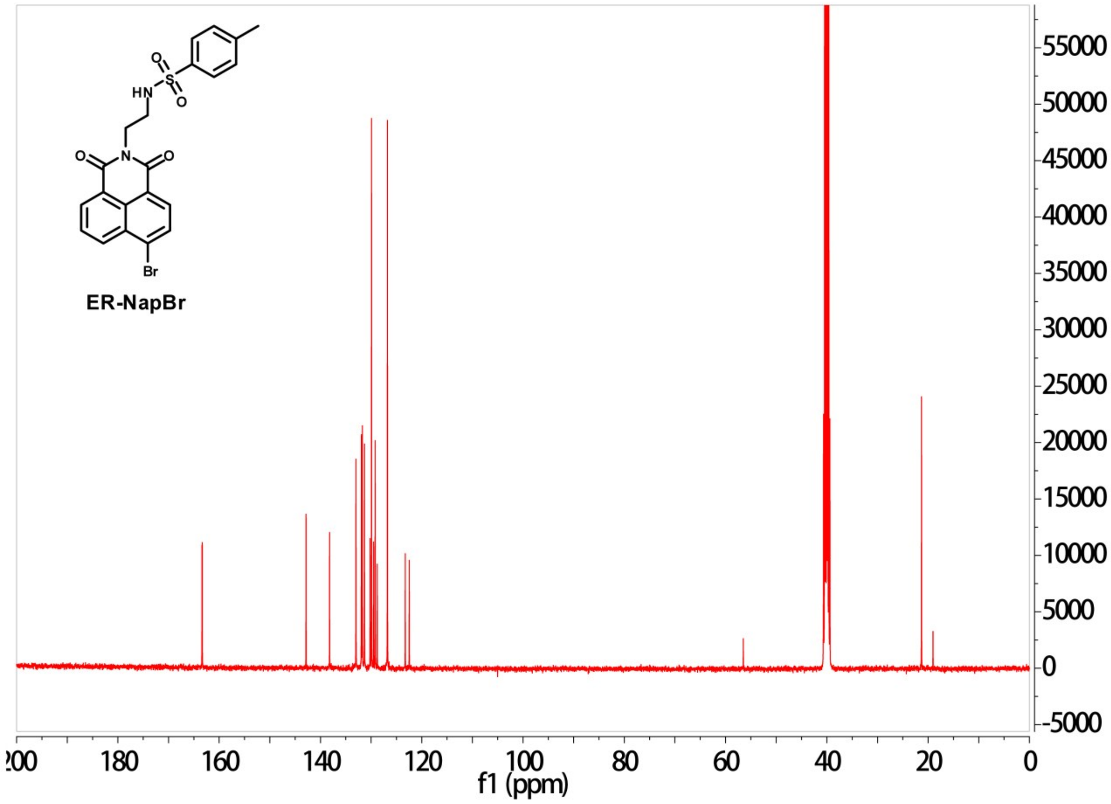
ER-NapBr
f1 (ppm)

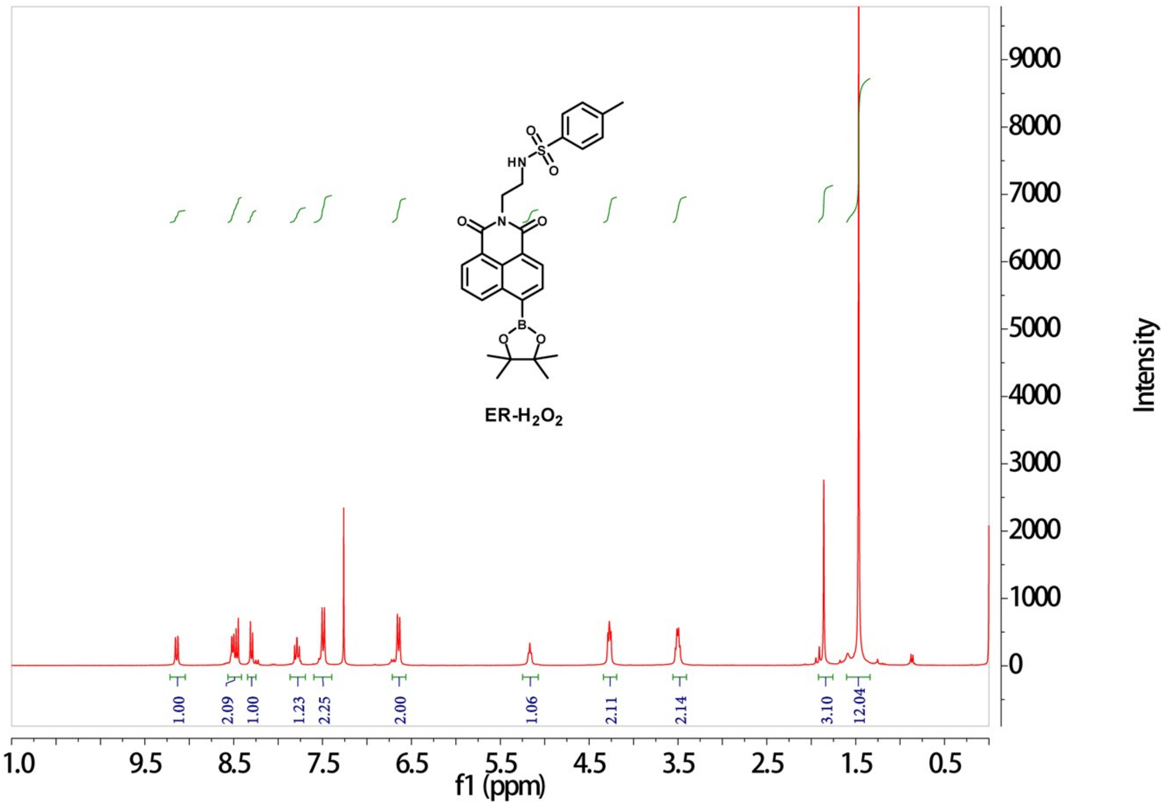
ER-H_2O_2
1.00
2.09
1.00
1.23
2.25
2.00
1.06
2.11
2.14
3.10
12.04
f1 (ppm)
Intensity

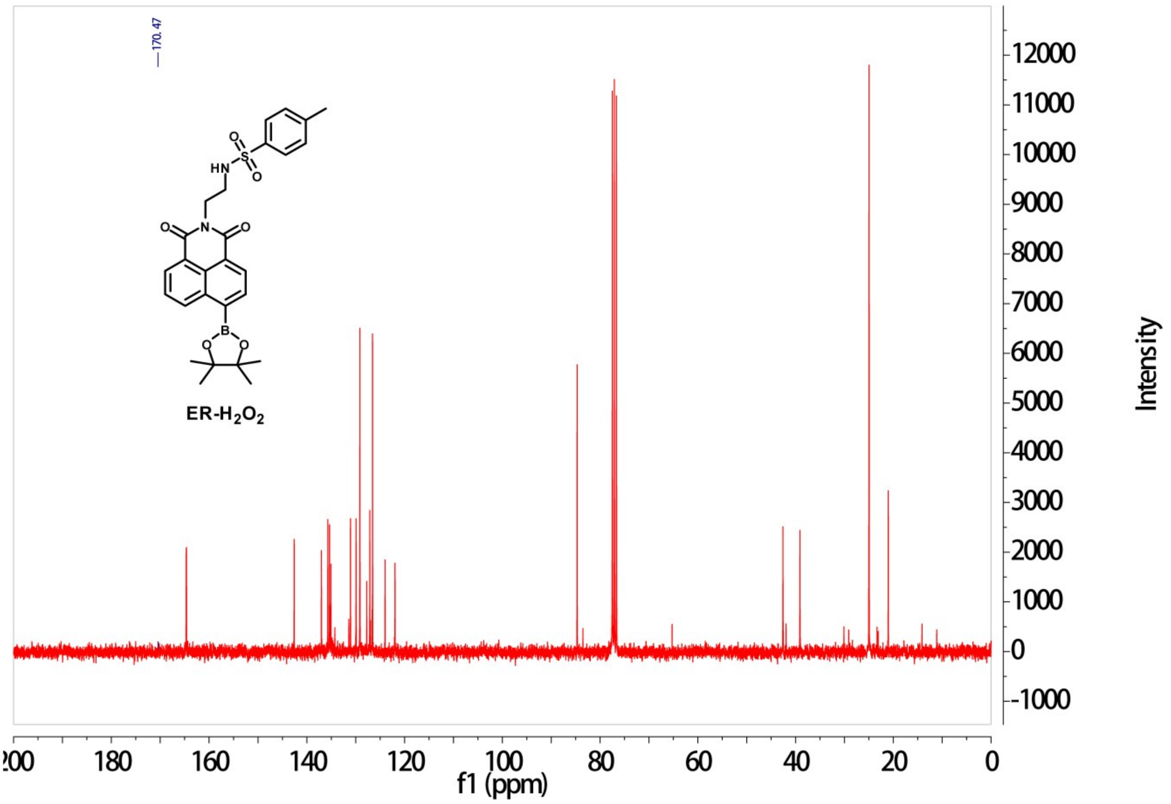
170.47
ER-H_2O_2
f1 (ppm)
Intensity

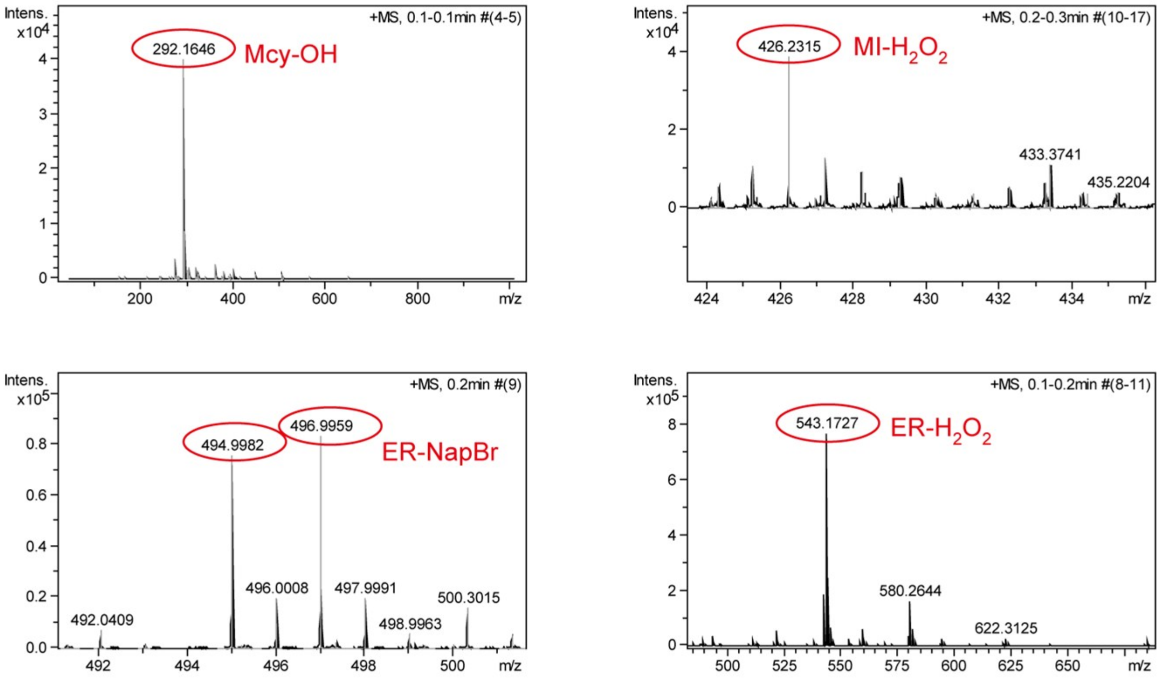
Intens.
x10^4
+MS, 0.1-0.1min #(4-5)
292.1646
Mcy-OH
m/z
Intens.
x10^4
+MS, 0.2-0.3min #(10-17)
426.2315
MI-H2O2
433.3741
435.2204
m/z
Intens.
x10^5
+MS, 0.2min #(9)
494.9982
496.9959
ER-NapBr
492.0409
496.0008
497.9991
498.9963
500.3015
m/z
Intens.
x10^5
+MS, 0.1-0.2min #(8-11)
543.1727
ER-H2O2
580.2644
622.3125
m/z
